# REP-1 deficiency induces aberrant mitochondrial metabolic rewiring from glycolysis to lipid oxidation in CHM disease

**DOI:** 10.1038/s41419-026-08592-6

**Published:** 2026-03-30

**Authors:** Sara Buonocore, Giuliana Giamundo, Chiara Barone, Iolanda Carratù, Giovanna Trinchese, Giovanni Andrea Vitale, Gianluca Fasciolo, Marcello Ziaco, Paola Venditti, Angelo Fontana, Maria Pina Mollica, Dario Antonini, Ivan Conte

**Affiliations:** 1 Department of Biology, University of Naples Federico II, Naples, Italy; 2 Institute of Biomolecular Chemistry ICB, National Research Council CNR, Pozzuoli, Italy

**Keywords:** Retina, Metabolic disorders

## Abstract

Choroideremia (CHM) is a hereditary retinal degenerative disorder characterized by progressive dysfunction of the retinal pigment epithelium (RPE) and photoreceptors with no available therapy. Despite the recognized genetic basis of CHM, the metabolic pathways driving disease progression remain poorly defined. By investigating REP-1 deficiency in CHM disease, our study reveals a previously unrecognized role for REP-1 in regulating GLUT-1 and GLUT-4 membrane trafficking, controlling glucose uptake, and reprograming mitochondrial metabolism toward lipid oxidation. This chronic metabolic shift results in reduced glycolytic flux, elevated oxidative stress, and compromised ATP production, culminating in a progressive retinal dystrophy. Notably, pharmacological restoration of GLUT trafficking via leptin administration re-established glucose uptake and mitochondrial function, rescuing cellular energetics both in vitro and in vivo. These findings establish REP-1 as a key regulator of retinal metabolic homeostasis and suggest that targeting glucose–lipid metabolic rewiring may represent a novel therapeutic strategy for CHM and related retinal dystrophies.

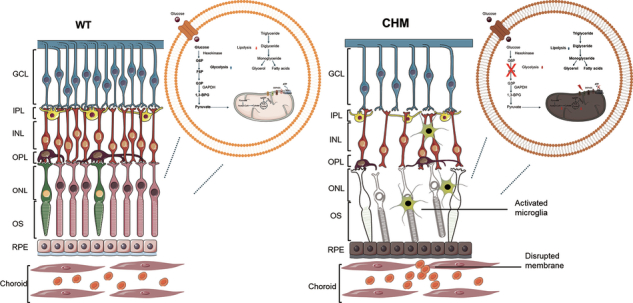

## Introduction

The retina, as part of the central nervous system, exhibits high energetic demand to sustain vision. Retinal neurons primarily utilize glucose, transported via GLUTs and metabolized through glycolysis and mitochondrial oxidation, with fatty acid β-oxidation serving as an alternative substrate under limited glucose availability [1]. Elevated β-oxidation increases cytoplasmic citrate, promoting pyruvate dehydrogenase kinase–mediated inhibition of pyruvate dehydrogenase, thereby impairing glucose uptake and oxidation. This metabolic interplay underscores the delicate balance between glucose and lipid utilization in maintaining retinal homeostasis. Disruption of this balance can induce hypoglycemia, lipotoxicity, inflammation, insulin resistance, and cell death, with chronic metabolic defects driving retinal dystrophies (RDs) [2, 3]. However, how the metabolic pathways contribute to the onset, progression or severity of the RDs has not been fully elucidated. Among these, Choroideremia is a rare X-linked chorioretinal dystrophy with an estimated prevalence of ~1 in 50,000 males worldwide [4], caused by mutations in the Choroideremia (CHM) gene encoding Rab escort protein-1 (REP-1), which results in altered lipid metabolism. Clinically, CHM patients manifest a progressive choroidal atrophy with degeneration of the retinal pigment epithelium, photoreceptors, and choroid [5], although the primary tissue affected remains unresolved [6]. In males, disease onset typically presents as nyctalopia in childhood, followed by gradual peripheral vision loss in adulthood and culminating in blindness at advanced stages [7]. Female carriers are usually asymptomatic but may exhibit mild phenotypic features, including fundus pigmentary changes and night blindness [8]. Notably, recent findings have shown that REP‑1–dependent prenylation is critical for Rab GTPase proteins, which are key regulators of intracellular vesicles formation, trafficking and fusion [9]. Interestingly, several Rabs are enriched in GLUT4‑containing vesicles, suggesting them in Glut4-enriched vesicle trafficking. Functional studies demonstrate that depletion of Rab8, Rab10 and Rab14 or expression of a prenylation‑deficient Rab4 form disrupts GLUT4 translocation to the plasma membrane [10]. Additionally, recent findings indicate that Rab activity also governs glycogen storage and ATP homeostasis via GLUT1 transport. Thus, Rab‑mediated compartmentalization of GLUT1 and GLUT4 at the cell surface may contribute to sustaining glucose supply and limiting lipid β‑oxidation as energy source [11]. However, the contribution of these pathways to retinal pigment epithelium and photoreceptor metabolism, and their pathological relevance in choroideremia, remains unresolved. Here, we demonstrate that REP 1 depletion compromises glucose uptake by impairing GLUT-1 and GLUT-4 expression and plasma membrane translocation, leading to mitochondrial dysfunction and reduced autophagic flux. In medaka, loss of REP 1 recapitulates CHM phenotype associated with impaired glucose uptake, altered lipid metabolism, and retinal degeneration. Notably, we show that pharmacological restoration of glucose uptake ameliorates retinal phenotype in CHM medaka model, establishing a mechanistic link between REP 1 deficiency, mitochondrial metabolism and retinal degeneration, and highlighting potential therapeutic strategies to counteract retinal degeneration in Choroideremia.

## Materials and methods

### Sex as a biological variable

We employed medaka larvae at stage 40, prior to gonadal maturation and external sex differentiation, as a model system. At this stage, sex is undetermined, and given that olRep‑1 resides on chromosome 14, the phenotype is expected to be independent of sex.

### RNA sequencing and computational analysis

Total RNA extraction from ARPE-19^WT^, Arpe-19^sh-REP1^ and Leptin-treated Arpe-19^sh-REP1^ cells was performed using the miRNeasy Kit (QIAGEN 217004) following the manufacturer’s instruction. The concentration of total RNA was measured using a NanoDrop spectrophotometer (Thermo Fisher), while its quality was evaluated with the TapeStation 4150 (Agilent Technologies). Briefly, 100 ng of RNA from each sample underwent mRNA isolation via polyT-coated beads, followed by indexed library preparation utilizing the Nextflex Rapid Directional RNA-Seq kit 2.0 Kit with PolyA An equimolar quantity of each DNA library was sequenced using the Illumina NovaSeq System (Illumina) with 2 × 150 bp paired-end, 30 M run. The resulting Fastq files containing reads were trimmed using Trim Galore! to remove low-quality sequences and adapters. Alignment was performed with RNA STAR (10.1093/bioinformatics/bts635) on the human h38 reference assembly. The expression levels of genes were determined with featureCounts (10.1093/bioinformatics/btt656), and the differential expression analysis was performed using DESeq2 (10.1186/s13059-014-0550-8). Differential expressed genes (DEGs) were filtered using the FDR ≤ 0.01 and Functional annotation analysis was carried out with DAVID and ShinyGO tools (10.1093/nar/gkac194; 10.1038/nprot.2008.211; 10.1093/bioinformatics/btz931). Heat maps and bubble profile plots were generated using respectively Morpheus (https://software.broadinstitute.org/morpheus) and with SRplot [12].

### Cell culture and treatments

ARPE-19 cell lines from ATCC were cultured as previously reported in ref. [13]. For Leptin and insulin treatments, cells were synchronized by serum deprivation for 16 h. We used a sub-confluent cell culture (i.e., 80% of confluence) for each in vitro experiment. Leptin stimulation was carried out with 150 nM of animal-free recombinant human Leptin (AF-300-27-200μg, Peprotech) for 3 h at 37 °C, 5% CO_2_ in a humified incubator. Insulin stimulation was assessed by using 1 µM of Insulin (I9278, SIGMA) for different time points at 37 °C, 5% CO_2_ in a humified incubator. Autophagic flux was evaluated as previously reported [14].

### Plasmids and transfections

Cells were transfected at 80% confluence with 1 μg of PCS2-7MYC-GLUT4-GFP (modified from Addgene 32751) for 24 h, using Lipofectamine 2000 (Invitrogen, 12566014), following the manufacturer’s protocol.

### Generation of shREP1 ARPE-19 cells

ARPE-19 cells expressing sh-REP-1 against the target sequence “CGGTATGGCAACACTCCATTT” were obtained by transducing WT with the lentiviral vector MISSION TRC2 pLKO.1-CMV-Neo at a concentration of 1 × 10^9^ particles/ml (TRCN0000065179 - Sigma Aldrich). A MISSION pLKO.1-puro non-Target shRNA Control Transduction Particles at ≥1 × 10^6^ particles/ml (SHC016V Sigma Aldrich) was used as a control.

### RNA extraction, retro-transcription, and real-time PCR

To evaluate CHM gene silencing, qRT-PCR was performed as previously described [14]. The qRT-PCR reactions were performed with nested Human REP-1 primers: Forward CTTCACTGTCTTGGCGGTAT; Reverse ATTCCACCAAACACAGCACAC and carried out with the on Real-Time PCR QuantStudio 5 System (Applied Byosistems). The qRT-PCR reaction was performed using cDNA (400 ng), 10 μl of SYBR Green Master Mix (Thermo Scientific), and 400-nM primers, in a total volume of 20 μl. Each sample was analyzed in triplicate, using HPRT (Forward primer GTTGGGCTTACCTCACTGCT, Reverse primer TCATCGCTAATCACGACGCT) expression levels as endogenous control. The PCR conditions were as follows: Preheating, 95 °C for 60 s; cycling, 45 cycles of 95 °C for 10 s, 60 °C for 10 s, and 72 °C for 15 s. Quantified results were expressed as cycle threshold (Ct). The Ct values were averaged for each triplicate. Differences between the mean Ct values of the REP-1 gene and those of the reference gene were calculated as DCtgene = Ctgene–Ctreference. Relative expression was analyzed as 2-DCt. Relative fold changes in expression levels were determined as 2-DDCt.

### Immunofluorescence

Cells and Medaka fish at stage 40 were fixed and analyzed as described in ref. [15]. The following primary antibodies, incubated overnight at 4 °C were used: anti-GFP (1:500 Invitrogen A6455), rabbit anti- GLUT1 (1:1000 Novus NB 300-666), rabbit mouse anti- Tom20 (1:500, Santa Cruz SC-136211), rabbit anti-Citrate Synthase (1:2000), rabbit anti-LAMP1 (1:400, Abcam ab24170), mouse anti-ZPR1 (1:100, Abcam ab174435) mouse anti-Rhodopsin (1:100, Invitrogen MA5-11741). All incubations were performed overnight. After washing with 1% PBS, slides and cells were incubated with the following secondary antibodies: Alexa 488 goat anti-rabbit/mouse (1:1000, Invitrogen A-11008 rabbit, A-11032 mouse), Alexa 594 goat anti-mouse/rabbit (1:1000, Invitrogen A-11032 mouse, A-11037 rabbit) and DAPI (1:500, Vector Laboratories H-1200) for 1 h at room temperature; then, the slides were washed with 1% PBS and mounted with PBS/glycerol and imaged with a Olympus confocal microscope (Olympus FV3000).

### Quantification of mitochondria morphology

Quantitative evaluation of morphological and network connectivity parameters of mitochondria obtained by Tomm20 and Citrate synthase immunofluorescences were performed using ImageJ/Fiji (Mito Analyzer Tool), as described in ref. [16]. 3D-images of mitochondrial morphology were obtained as described in ref. [16]

### Western blot analysis

WBs were performed and analyzed as previously described in ref. [15]. Medaka embryos at stage 24 were lysed and analyzed as previously described [15]. Both protein concentrations were determined by Bradford colorimetric assay and quantified using a spectrophotometer (UV-3100PC spectrophotometer, VWR). For Western blot analysis, the following primary antibodies were used: mouse anti-REP1 (1:500, Santa Cruz SC-23905), rabbit anti-β actin (1:1000, Cell Signaling 4967), rabbit anti- Insulin Receptor (1:1000 Abclonal A19067), rabbit anti-IRS1 (1:1000, Abclonal A0245), rabbit anti-PI3 Kinase p110 (1:1000 Abclonal A0982), rabbit anti- PI3 Kinase p85 (1:1000, Abclonal A11177), rabbit anti-phospho-Akt (Ser473) (1:1000, Cell Signaling 4060), rabbit anti-Akt (1:1000, Cell Signaling 9272), rabbit anti- GSK3β (1:1000, Abclonal A11731), rabbit anti- GLUT1 (1:1000 Novus NB 300-666), mouse anti- GLUT4 (1:500 Santa Cruz SC-53566), rabbit anti-Citrate synthase (1:1000, Invitrogen PA5-22126), mouse anti-Tomm20 (1:500, Santa Cruz SC-136211), rabbit anti-LC3 (1:1000, Novus NB100-2220), mouse anti-total OXPHOS cocktail (1:500 Abcam ab110413). Secondary antibody: goat anti-rabbit IgG antibody, HPR conjugate, and goat anti-mouse IgG antibody HPR conjugate (1:10000 EMD Millipore, 12-348; 12-349). Full and uncropped western blots were uploaded as Supplementary Material.

### Live assay

1 × 10^5^ cells/well were seeded in a 35 mm imaging dish with a glass bottom (Ibidi, 81158). Cells were washed in DMEM without Phenol Red (Gibco A14430-01) and treated with the following probes:

*Glucose uptake* was monitored using the fluorescent deoxyglucose analog 2 NBDG (Thermo Fisher Scientific, N13195). ARPE 19 cells were incubated with 0.25 mM 2 NBDG for 30 min. Uptake was quantified as mean fluorescence intensity (IntDen/Area). For medaka assays, stage 40 larvae were exposed to 0.6 mM 2 NBDG in Yamamoto medium for 5 h, rinsed three times for 20 min, and imaged using a Leica MZ10F microscope.

*CM-H2DCDFDA assay*. Briefly, cells were treated with 5 μM CM-H2DCDFDA (Invitrogen, C6827) for 30 min. After washing thrice with PBS, the fluorescence intensity was acquired by confocal microscopy Data were expressed as mean fluorescence intensity (IntDen/Area). Medaka fish at stage 40 were incubated in 10 μM CM-H2DCDFDA for 3 h, and the fluorescence intensity of larvae was evaluated using Leica (MZ10F).

*MitoSOX*. Mitochondrial superoxide generation was evaluated in live cells using MitoSOX (Invitrogen, 36008), a fluorogenic reagent specifically targeted to mitochondria. Cells were incubated at 37 °C in the dark for 30 min in Phenol Red-free DMEM-F12 containing 5 μM MitoSOX. Following the removal of excess dye by washing with PBS and quantitative measurements of red fluorescence were determined using ImageJ by measuring mean fluorescence signal intensity (calculated as IntDen/Area).

*Mitotracker Deep Red* Mitochondria were evaluated using Mitotracker Deep Red FM (Invitrogen, M22426). ARPE‑19 cells were incubated with 0.25 mM Mitotracker in DMEM‑F12 without phenol red at 37 °C in the dark for 1 h. For in vivo assays, stage‑40 medaka larvae were exposed to 1 mM Mitotracker in Yamamoto medium for 5 h.

*LipidSpot 488* (Biotium, NC1669425) was performed on cells following the manufacturer’s instructions. After acquisition of the signal by the Olympus FV3000 Confocal, images were analyzed with ImageJ and quantification of the lipid spots signal was represented as the ratio of Green-positive stained cells to Hoechst-stained nuclei.

*C11-BODIPY 581/591* (Invitrogen, D3861) was performed to evaluate lipid peroxidation on ARPE-19 cells. Briefly, cells were treated for 30 min with 5 μM of C11-BODIPY 581/591. After three washing with PBS. Red and green fluorescence were measured using ImageJ as mean values to define fluorescence signal intensity (IntDen/Area). Then, quantification of lipid peroxidation was calculated as the ratio of green to red values.

In all assays, Hoechst was used as a nuclear stain, and images were acquired by confocal microscopy (Olympus FV3000) and analyzed using ImageJ.

### Transmission electron microscopy

For standard electron microscopy (EM) analysis, cells were fixed in 2% glutaraldehyde (GA) prepared in 0.2 M HEPES buffer (pH 7.4) at room temperature (RT) for 30 min. Post-fixation was carried out using 1% osmium tetroxide for 60 min, followed by treatment with 0.25% uranyl acetate overnight. Dehydration was achieved through a graded ethanol series, and the samples were embedded in EPON resin under control conditions: overnight at 37 °C, 1 day at 45 °C, and an additional day at 60 °C. Ultrathin sections of 60 nm were precisely cut parallel to the substrate and placed onto a 200-mesh copper grid. Bright-field TEM imaging was performed on the dried sample utilizing an FEI TECNAI G2 200 kV s-twin microscope operating at 120 kV (Thermo Fisher Scientific, Waltham, USA). Digital images were captured using an Olympus VELETA camera.

### Seahorse analysis

Mitochondrial metabolism in ARPE-19 shREP1 cells and controls was assessed by the Seahorse XF24 analyzer (Seahorse Biosciences, North Billerica, MA, USA), by using the Cell Mito Stress Test kit (Agilent, Santa Clara, CA, USA, cat# 103015-100). Cells (3.0 × 10^5^/well) were seeded in DMEM‑F12 medium for 24 h, then transferred to a buffered base medium (Agilent Seahorse-103575) supplemented with 2 mM glutamine, 1 mM pyruvate and 25 mM glucose at pH 7.4. Basal respiration was calculated as in ref. [15]. Spare respiratory capacity (SRC) is the capacity of the cell to respond to an energetic demand and was calculated as the ratio between maximal respiration and basal respiration × 100. Mitochondrial respiration was expressed as the oxygen consumption rate per minute normalized to the number of cells. The mitochondrial respiration was also measured after the acute injections of fuel pathway inhibitors: UK5099 (2 µM), an inhibitor of the glucose oxidation pathway, blocking the mitochondrial pyruvate carrier; BPTES (2 µM), an inhibitor of the glutamine oxidation pathway, through inhibition of glutaminase; Etomoxir (4 µM), an inhibitor of long chain fatty acid oxidation, blocking the activity of carnitine palmitoyl-transferase 1. In our experimental conditions, the same cell number/well was plated before the OCR measurements; the cell count was obtained by using the Burker chamber. Real-time measurements of OCR were also normalized to total protein content using a Bradford assay.

### ATP determination assay

The intracellular ATP levels were measured using the ATP Determination kit (Molecular Probes; Invitrogen A22066) in a microplate reader for luminescence (Synergy™ HTX Multi-Mode Microplate Reader, BioTek), following the manufacturer’s instructions.

### Activity of ETC complexes

The activity of the complexes I, II, III of the electron transport chain (ETC) was determined spectrophotometrically [17] using a multi-mode microplate reader (Synergy™ HTX Multi-Mode Microplate Reader, BioTek). Activity of the complex IV (Chitochrome Oxidase, COX) was determined polarographically [18] using a Hansatech respirometer (Hansatech Instruments Ltd, Narborough Road, Pentney King’s Lynn, Norfolk PE32 1JL, United Kingdom).

### Medaka stocks and mo-injections

The Cab-strain of wild-type Medaka fish (*Oryzias latipes*) was maintained following standard conditions (i.e., 12 h/12 h dark/light conditions at 27 °C). Embryos were staged according to ref. [19]. A morpholino (Mo; Gene Tools LLC, Oregon, USA) was designed against the (Mo-*Ol-REP1*: 5′-G TG CTG AGA CAC GGT CAC GTG ACC A-3′) of the medaka orthologous of the *CHM* gene. Mo-Ol-REP1 was injected at 0.21 mM concentration into one blastomere at the one/two-cell stage. Importantly, Medaka fish has emerged as a powerful model system for studying retinal physiology and pathology. Despite evolutionary distance, the medaka retina shares striking similarities with the human retina in terms of cellular composition, laminar organization, and functional circuitry [20].

#### Study approval

All studies on fish were conducted in strict accordance with the Institutional Guidelines for animal research and approved by the Italian Ministry of Health, Department of Public Health, Animal Health, Nutrition and Food Safety in accordance with the law on animal experimentation (D. Lgs. 26/2014).

### Leptin treatments on medaka fish

For leptin treatment, at stage 24, eggs were digested for 3 h in agitation with 20 μg/ml of proteinase K and washed 5 times with ddH_2_O. Then, eggs were kept for 30’ in 10 mg/ml of Pronase and subsequently rinsed 5 times with ddH_2_O. Lastly, for the chorion dissolution, eggs were incubated in hatching enzyme for 2 h at 27 °C. Stage 24 larvae without chorion were treated overnight with 150 nM of Leptin until they reached stage 40.

### Lipid extraction and LC-MS/MS analysis

Three biological groups were analyzed: WT, MO-REP1, and MO-REP1 + leptin (*n* = 5 per group, each replicate a pool of 8 medaka). Samples were homogenized in 100 μL methanol, and lipids were extracted using the Bligh and Dyer method [21]. Extracts were resuspended in 500 μL LC-MS grade methanol and analyzed on a Vanquish UHPLC system coupled to a Kinetex 2.6 μm EVO C18 column (50 × 2.1 mm, 100 Å; Phenomenex) and an Orbitrap Exploris 240 mass spectrometer with a heated electrospray ionization source. Five microliters of each sample were injected, and chromatographic separation was performed at 0.5 mL/min using mobile phase A (H₂O, 0.1% formic acid) and mobile phase B (ACN, 0.1% formic acid). The gradient started at 50% B, increasing linearly to 99% B over 15 min, followed by a washing phase at 99% B, and then re-equilibration to the initial conditions.

The HESI source was used in positive mode and set as follow: auxiliary gas flow = 5 AU, sweep gas flow = 2 L/min, and sheath gas flow = 45 AU, ion transfer tube temperature = 325 °C, vaporizer temperatures = 300 °C, spray voltage = 3.5 kV. Scan range = 150–1500 *m*/*z*, MS1 resolution = 45 K. The analysis was performed in Data Dependent Acquisition mode, 5 most abundant ions were fragmented, MS2 resolution = 30,000, isolation window = 1 *m*/*z*, stepped normalized collision energies = 15, 35, and 45 eV. Dynamic exclusion was applied after 3 occurrences for 5 s. The .raw files were converted to.mzml format using Msconvert from ProteoWizard [22] and processed with MZmine4 (Version 4.3) [23]. The output included an .mgf file with MS data, a .csv file containing the quantification table, and additional .csv file detailing the connections between different ion forms for each molecule [24]. A metadata table (.csv) was also generated, containing experimental information about the sample groups to integrate the data. All the files were uploaded to GNPS2 [25] and analyzed through the Feature-Based Molecular Networking workflow [26]. Metabolites annotation was carried out both via the Lipid annotation module in MZmine4, and GNPS2 library matching, and finally manually curated (Table S3 and Supplementary Fig. 6). For the statistics, the quantification and the metadata tables were uploaded to FBMN-STATS, a web site for the downstream statistical analysis of Feature-Based Molecular Networking (FBMN) results [27]. Before statistical analysis, data were subjected to blank removal, TIC normalization, and imputation. Significance was calculated via one-way ANOVA, followed by Tukey’s post hoc test for pairwise comparisons, which includes adjustment for multiple comparisons. The Benjamini–Hochberg false discovery rate (FDR) was employed (FDR-adjusted *p* < 0.05). The Molecular Networking and library search output is publicly available at the GNPS2 job: https://gnps2.org/status?task=732b607a52d945188e86c3fde1809107. Raw data are available on Zenodo at the following link: https://zenodo.org/records/15609818.

### Statistics

All data are expressed as mean ± standard error, unless otherwise specified. Statistical analyzes were performed on at least three independent experiments for each experimental condition. In all experiments, statistical significance of differences between groups was assessed using the *t*-test. The corresponding *p*-values are reported in figure legends.

## Results

### REP-1 deficiency alters GLUT4 membrane translocation

**Fig. 1:**
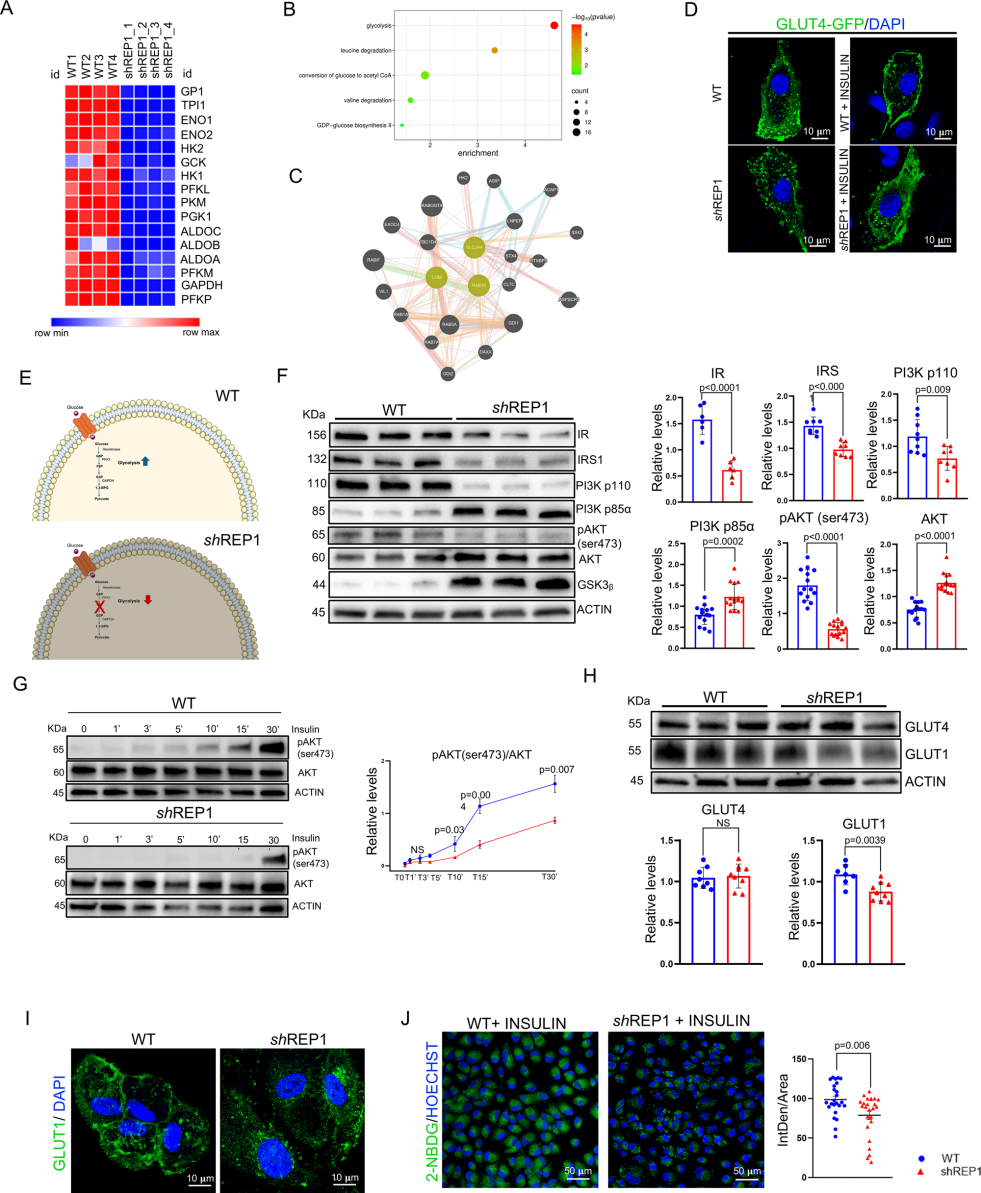
The absence of REP-1 affects GLUT4 trafficking to the plasma membrane, impairing glucose uptake. **A** Heat map representing the expression of the 16 genes associated with Glycolysis in ARPE-19 WT and ARPE-19^shREP1^ cells. **B** Bubble plot representing the functional annotation analysis of the downregulated genes in Arpe-19sh-REP1 compared to WT. Counts indicate the number of genes found in each process. Color indicates the scale for the −log10 (*p*value). **C** Physical interactions, generated by GeneMANIA, highlight REP-1, RAB10 and GLUT4 binding. **D** Representative immunofluorescence image of GLUT4-GFP and its translocation to the plasma membrane in basal and insulin-stimulated ARPE-19 WT and ARPE-19^shREP1^. Cells were transfected with GFP-tagged GLUT4. After 24 h cells were stimulated with Insulin (1 μM; 30’), fixed and analyzed by confocal microscopy. Nuclei were counterstained with Dapi. Scale bar 10 μm. **E** Schematic comparison of glycolysis metabolism in WT and REP-1 deficiency condition. In WT, GLUT4 guarantees glucose uptake in cytoplasm. When REP-1 is ablated, GLUT4 fails to reach the membrane efficiently, leading to impaired glucose uptake. **F** Representative images of Western Blot analysis of insulin receptor (IR), insulin receptor substrate 1 (IRS1) and some members of its signal transduction network PI3K p110, PI3K p85α, pAKT (ser473), AKT and GSK3β. β-Actin was used as loading control. Graphs show the levels of IR, IRS1, PI3K p110, PI3K p85 α, GSK3β and pAKT (ser473)/AKT ratio ± SEM (*n* = 3 experiments at least). Statistical test: unpaired *t*-test. **G** Representative western blot of time course of insulin-induced phosphorylation of AKT in ARPE-19 WT and ARPE-19^shREP1^ cells. Cells were treated with 1 µM of insulin in parallel at various time points for 30 min. β-Actin was used as loading control. Graph shows the mean of pAKT(ser473)/AKT ratio ± SEM (n = 3 experiments at least). Statistical test: unpaired *t*-test. **H** Immunoblots and calculated levels of GLUT1 and GLUT4. Data are expressed as value ± SEM of GLUT1 and GLUT4 relative to β-Actin, used as loading control. (*n* = 3 experiments at least). Statistical test: unpaired *t*-test **I** Immunofluorescence labeling images of GLUT1 (green) and DAPI (blue) in ARPE-19 WT and ARPE-19 ^shREP1^. Scale bar 10 µm. **J** Representative confocal images of 2-NBDG in ARPE-19 WT and ARPE-19 ^shREP1^ for glucose uptake evaluation after insulin stimulation (1 µM). Nuclei were counterstained with HOECHST (blue). Scale bar 50 µm. Graph shows the single values of the normalized fluorescence intensity (IntDen/Area) of 2-NBDG-positive cells ± SEM (*n* = 3 experiments at least). Statistical test: unpaired *t*-test.

Given the role of Rab escort protein-1 (REP-1) in intracellular vesicle trafficking and metabolic pathways, we evaluated the consequences of REP-1 loss of function in CHM by using integrated comparative analysis on unbiased RNA-seq of ARPE-19 cells silenced for REP-1 (hereafter termed ARPE-19^sh-REP1^; Supplementary Fig. 1A, B). We identified 5186 differentially expressed upregulated genes and 5071 downregulated genes (FDR ≤ 0.01) in ARPE-19^shREP1^ cells compared to controls. Notably, gene ontology and functional annotation clustering analyzes identified lipid metabolism as an upregulated category by REP-1 silencing. Moreover, a significant downregulation was also observed for a set of key genes involved in glycolysis metabolism (Fig. 1A, B), like previous observations in zebrafish-derived samples [28]. These data suggested that a transcriptional mechanism might mediate a metabolic switch from glucose to lipid when ARPE-19 cells were depleted of REP-1 gene. Considering that REP-1 is a key component of the Rab geranylgeranyl transferase complex, playing a central role in the post-translational modification and activation of Rab GTPases by facilitating their membrane targeting [29, 30] and role in vesicle trafficking, we hypothesized that the altered cell metabolism might depend on an altered localization and function of one or more Rab proteins. Interestingly, among the Rab GTPases, Ras-related protein Rab-10 appeared particularly attractive to explain the metabolic dysfunction because it had been previously shown to control insulin-mediated Glucose Transporter Type 4 (GLUT4) trafficking to the plasma membrane, which facilitates the uptake of glucose into cells in response to insulin, ensuring glycolysis to meet the energy needs of the cell. Thus, we explored potential similarities in altered gene profile between 3T3-L1^RAB10-KO^ [31] and ARPE-19^sh-REP1^ cells. Notably, comparison of gene expression profiles from 3T3-L1^RAB10-KO^ and ARPE-19^sh-REP1^ cells revealed shared gene expression patterns, with 25% of the altered genes in 3T3-L1^RAB10-KO^ showing concordant alterations in ARPE-19^sh-REP1^ cells. Interestingly, the common DEGs are enriched in lipid and mitochondrial metabolism (Tables S1 and S2). These data suggest a common metabolic dysregulation in 3T3-L1^RAB10-KO^ and ARPE-19^sh-REP1^ cells, which is consistent with the possibility that the perturbation of GLUT4 trafficking in ARPE-19 mediates glycolysis alteration in CHM phenotype. Remarkably, RAB10 was experimentally shown to interact with CHM by medium-confidence data from affinity chromatography and co-immunoprecipitation assays (BioGRID; IntAct). Furthermore, gene network based on physical interaction revealed a REP-1/RAB10 axis as a possible GLUT4 protein partner (Fig. 1C). Thus, if most of the changes in gene expression pattern, caused by REP-1 depletion, are mediated by dysfunctional RAB10, trafficking of GLUT4 endosomal vesicles to the plasma membrane should be inhibited upon insulin stimulation. Notably, insulin administration was not sufficient to induce translocation of GLUT4 from intracellular vesicles to the plasma membrane in anterograde transport in ARPE-19^sh-REP1^ compared to control cells (Fig. 1D, E and Movies S1 and S2). Under normal conditions, insulin stimulates the PI3K/Akt signaling pathway, leading to GSK3-β inhibition. This allows GLUT4 transporters to relocate to the cell membrane, facilitating glucose uptake. However, when GSK3-β is upregulated, it can disrupt this process. Therefore, we hypothesized that the defective translocation of GLUT4 could be further due to altered insulin signaling in ARPE-19^si-REP1^cells. Consistently, we noted that depletion of REP-1 in ARPE-19 cells reduced the expression of Insulin Receptor (IR) and Insulin Receptor Substrate 1 (IRS1) accompanied by a reduction of PI3K substrate P110 and phopsho-AKT and an increase of PI3K substrate P85, and GSK3-β (Fig. 1F), further supporting that GLUT4 membrane translocation and glucose homeostasis could be compromised for the insulin insensitivity. Coherently, the insulin time course from 0 to 30’ is not able to rescue phosphorylation of AKT in ARPE-19^sh-REP1^cells compared to control (Fig. 1G). Interestingly, previous studies have shown that in RPE, GLUT4 is the main glucose transporter and GLUT1 plays a marginal role. However, in several different tissues an increase of GLUT1 expression can compensate for the absence of GLUT4 in the uptake of glucose [32]. Therefore, we checked whether expression of GLUT1 was modified in ARPE-19^sh-REP1^ cells. Notably, the expression of GLUT1 was reduced, but not GLUT4, which only resulted in mislocalized (Fig. 1H, I). Importantly, this phenotype was associated with a defective uptake of glucose in ARPE-19^si-REP1^ cells (Fig. 1J). Altogether, these data suggest that REP-1 controls glucose transporter localization and expression possibly by modulating the RAB GTPase activity.

### REP-1 deficiency drives mitochondria dysfunction

**Fig. 2:**
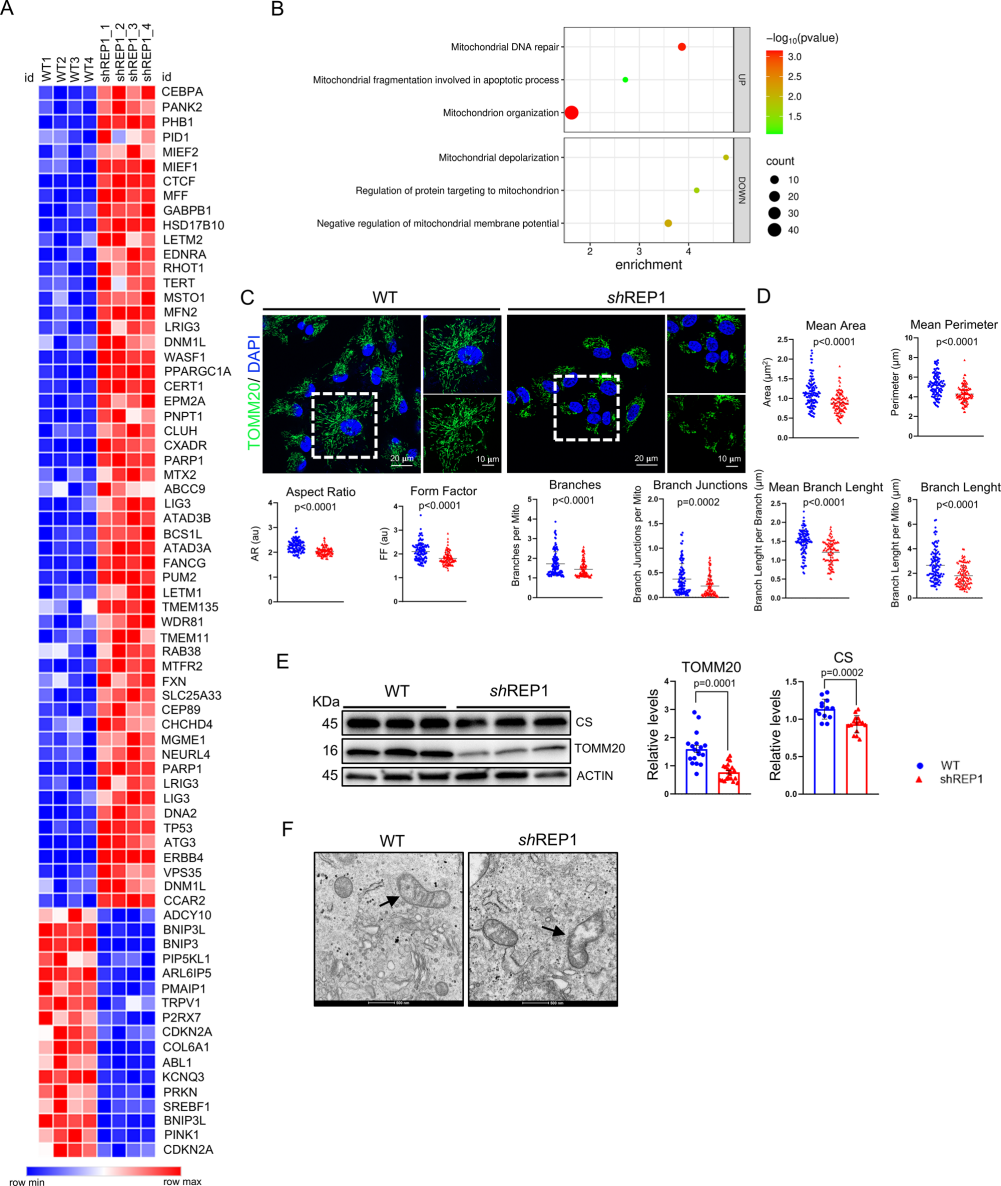
REP-1 deficiency drives alteration in mitochondrial morphology and function. **A** Heat map representing the expression of the DEGs genes associated to mitochondrial activity in ARPE-19 WT and ARPE-19^shREP1^ cells. **B** Bubble plot representing the functional annotation analysis of up- and downregulated genes in Arpe-19sh-REP1 compared to WT. Counts indicate the number of genes found in each process. Color indicates the scale for the −log10 (*p*value). **C** Confocal images of TOMM20 (green) immunofluorescence on ARPE-19 WT and ARPE-19^sh-REP1^ cells. Scale bar 20 µm. Magnified box scale bar 10 µm. **D** Quantitative analysis of morphological parameters and network connectivity of mitochondria in ARPE-19^shREP1^ compared to ARPE-19 WT cells. Results are shown as values ± SEM; Experimental replicates=at least 3; Statistical analysis: Student’s *t*-test. **E** Representative western blot image and calculated levels (right) of TOMM-20 and Citrate synthase. Data are expressed as values ± SEM of TOMM-20 and Citrate synthase relative to β-Actin, used as loading control. (*n* = 3 experiments at least). Statistical test: unpaired *t*-test. **F** Representative electron microscopy images of mitochondria in ARPE-19 WT and shREP1 cells, highlighting alteration of mitochondrial cristae in absence of REP-1.

**Fig. 3:**
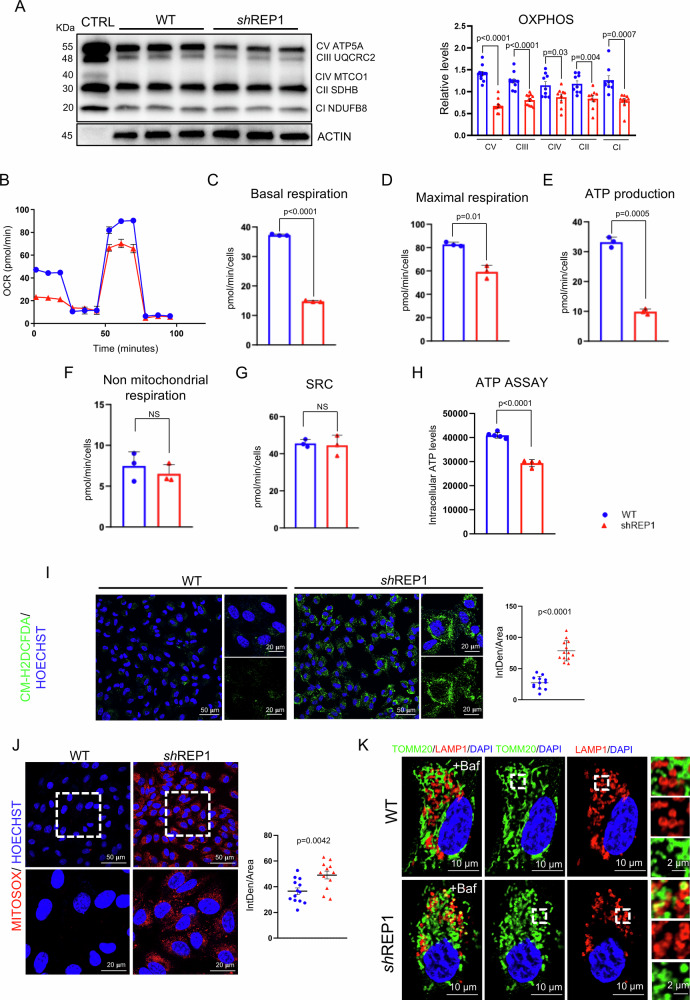
Mitochondrial dysfunction in ARPE-19 ^shREP1^. **A** Representative Western blot analysis of OXPHOS mitochondrial subunits performed with an antibody cocktail specific for representing the five mitochondrial oxidative phosphorylation complexes on ARPE-19 WT and ARPE-19^shREP1^ total cellular protein extracts. CTRL (rat heart tissue lysate) is a positive control supplied with the cocktail. The histogram on the right represents the mean ± SEM of the ATP5A, UQCRC2, SDHB, MTCO1 and NDUFB8 protein expressions levels relative to β-Actin, used as loading control (*n* = 3 experiments). Statistical test: unpaired *t*-test. Representative graph of Cell Mito Stress assay performed by Seahorse XF analyzer in ARPE-19 WT and ARPE-19^shREP1^ cells is reported. In the bar charts (**B**), each point in the OCR time courses is the average of three technical replicates. The values of Basal respiration (**C**), maximal respiration (**D**), ATP production (**E**), non-mitochondrial respiration (**F**) and spare respiratory capacity (**G**) are expressed as means ± SEM. Statistical analysis: unpaired *t*-test. **H** Intracellular ATP levels in ARPE-19^shREP1^ compared to ARPE-19 WT, measured using ATP determination kit. Graph show mean ± SEM of at least three independent measurements. Statistical analysis: unpaired *t*-test. **I** Demonstrative confocal images of intracellular ROS in ARPE-19 WT and ARPE-19^shREP1^ cells determined using the fluorescent probe CM-H2DCFDA (green). Nuclei were counterstained with HOECHST (blue). Scale bar 50 µm. Magnified box 20 µm. Data represent single values of fluorescence intensity (IntDen/Area) ± SEM (*n* = 3 experiments at least). Statistical test: unpaired *t*-test. **J** Representative images of MitoSOX (red), in ARPE-19 WT and ARPE-19^shREP1^ cells. Nuclei were counterstained with HOECHST (blue). Scale bar 50 µm. Magnified box 20 µm. Data represent single values of the normalized fluorescence intensity (IntDen/Area) ± SEM (*n* = 3 experiments at least). Statistical test: unpaired *t*-test. **K** Representative confocal images of ARPE-19^shREP1^ and WT cells, treated with Baf A1, immunolabeled with anti-TOMM20 (green), LAMP-1 (red) antibodies and nuclei were counterstained with DAPI (blue). Scale bar 10 µm. Magnified box 2 µm.

Defects in the glucose uptake disrupt glycolysis and consequently might trigger dysfunctional mitochondrial metabolism. Therefore, to understand the functional significance of the altered glucose uptake induced by REP-1 depletion, we first analyzed DEGs from RNA-SEQ, observing several genes involved in mitochondrial activity (Fig. 2A, B). Next, we analyzed whether change in glucose metabolism was associated with alterations in mitochondrial network architecture in ARPE-19^si-REP1^ compared to control cells. We used TOMM20 immunostaining to label mitochondria. Notably, mitochondrial network in each ARPE-19^sh-REP1^ cell was significantly reduced compared to control (Fig. 2C). Measurement for area and perimeter, showed a statistically significant reduction of mitochondrial structure (Fig. 2D). Accordingly, mitochondrial shape also resulted in highly altered form factor (FF) and aspect ratio (AR) parameters. Furthermore, the analysis of the mitochondrial network revealed a reduction in overall connectivity and morphological complexity. This was evident through a decrease in the number of branches, branch junctions, and the total accumulated length of branches within the skeletonized network (Fig. 2D). These changes highlight significant alterations in mitochondrial morphology. We confirmed this observation by quantifying MitoTracker labeling and CS immunostaining (Supplementary Fig. 1C–F), indicating a further reduction in overall network complexity associated with an altered morphology. Notably, these changes were also accompanied by a reduction in TOMM20 expression level (Fig. 2E), suggesting that morphological alteration might be associated with dysfunctional mitochondrial respiratory activity. In support of this hypothesis, morphological examination using transmission electron microscopy (TEM) confirmed a dramatic alteration of mitochondrial cristae, where respiratory chain complexes are situated (Fig. 2F). Notably, this phenotype was accompanied by a significant alteration of OXPHOS protein expression in ARPE-19^sh-REP1^ compared to control cells (Fig. 3A). The protein levels of NDUFB8 (complex I), SDHB (Complex II), UQCRC2 (complex III), MTCO1 (complex IV), and ATP5A (Complex V) decreased in absence of REP-1 in ARPE-19 cells, further supporting a dysfunctional mitochondrial respiratory activity. Thus, we investigated possible changes in mitochondrial respiration by measuring mitochondrial complex activities on isolated mitochondria of ARPE-19^sh-REP1^ compared to control cells. Notably, mitochondrial complex I–II and complex IV activities were significantly decreased, whereas the activity of complex III was not affected in ARPE-19^sh-REP1^ compared to control cells, respectively (Supplementary Fig. 1G). We confirmed this observation by testing the oxygen consumption rates (Fig. 3B). Notably, we found a significant reduction in basal (Fig. 3C) and maximal (Fig. 3D) respiratory rate in ARPE-19^sh-REP1^ compared to control cells, indicating a lowering of mitochondrial oxidative capacity. Consistently, the ATP production was also significantly reduced in ARPE-19^sh-REP1^ compared to control cells (Fig. 3E), whereas no difference was found in non-mitochondrial respiration and SRC (Fig. 3F, G). In accordance, we detected a reduction in intracellular ATP level (Fig. 3H). Considering that a defective mitochondrial ETC may lead to a higher likelihood of electrons leaking and reacting with oxygen to produce excessive reactive oxygen species (ROS), we examined whether depletion of REP-1 would induce an increase of ROS. Notably, measurement of the fluorogenic probe 2’,7’-dichlorodihydrofluorescein diacetate (H_2_DCFDA) that, is converted into a highly fluorescent form called 2’,7’-dichlorofluorescein (DCF) in the presence of ROS, showed a high increase of fluorescent intensity of DCF in ARPE-19^sh-REP1^ compared to control cells (Fig. 3I), suggesting that dysfunctional mitochondria produced ROS. To ensure that these alterations were dependent by dysfunctions of mitochondria, we used a highly selective fluorescent dye (Mitosox) to detect mitochondria-derived ROS. Consistently, we observed that the intensity of Mitosox staining was significantly increased in ARPE-19^sh-REP1^ compared to control cells (Fig. 3J), suggesting a high levels of ROS within the mitochondria of ARPE-19^sh-REP1^. Interestingly, a burst of ROS production within mitochondria is demonstrated to be involved in the signaling for mitophagy [33]. Consistently, we observed a selective clearance of damaged mitochondria in ARPE-19^sh-REP1^ cells (Fig. 3K). However, the autophagy pathway was slightly reduced (Supplementary Fig. 1H) as previously reported.

### Depletion of REP-1 unbalances lipid metabolism in RPE

**Fig. 4:**
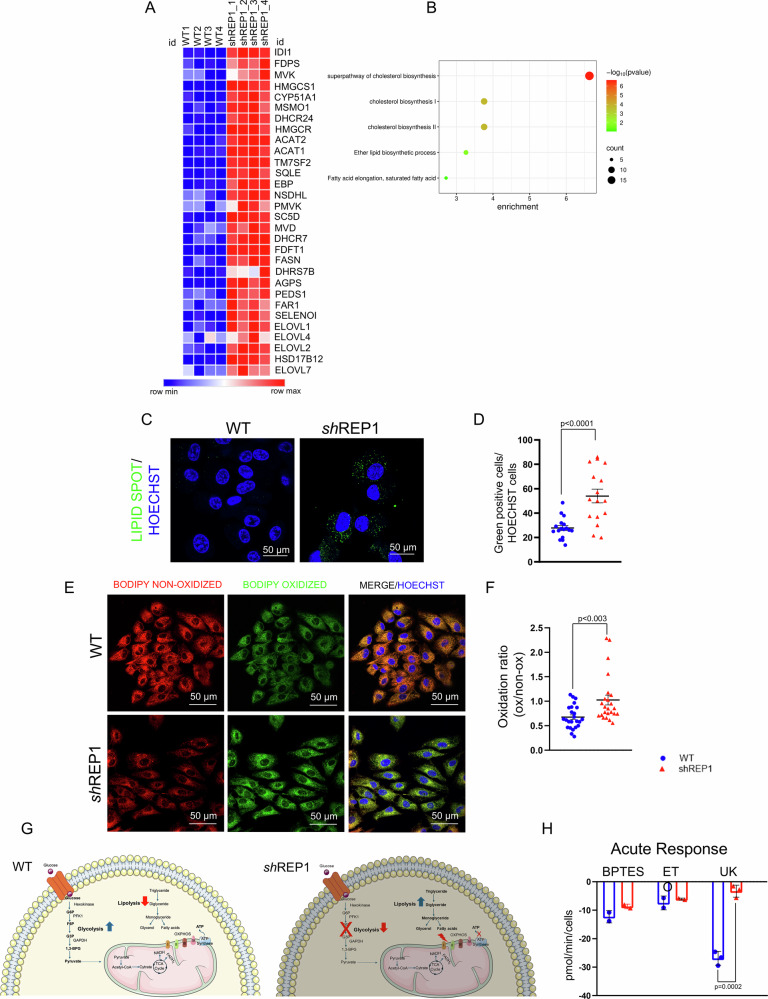
Depletion of REP-1 disrupts lipid metabolism. **A** Heat map representing the expression of the genes associated to lipid metabolism ARPE-19 WT and ARPE-19^shREP1^ cells. **B** Bubble plot representing the functional annotation analysis of the upregulated genes in Arpe-19sh-REP1 compared to WT. Counts indicate the number of genes found in each process. Color indicates the scale for the −log10 (*p*value). **C** Representative confocal images of ARPE-19 WT and ARPE-19^shREP1^ cells live stained with Lipid Spot (green) and HOECHST (blue). Scale bar: 50 µm. **D** Quantification of Lipid Droplets. Data are expressed as values ± SEM of the ratio of cell positive for lipid spot to HOECHST positive cells. (*n* = 3 experiments at least). Statistical test: unpaired *t*-test. **E** Representative confocal images of Lipid peroxidation evaluated using BODIPY C11 fluorescence in ARPE-19 WT and ARPE-19^shREP1^ cells. Nuclei are counterstained with HOECHST. Scale bar 50 µm. **F** Quantification of lipid peroxidation. Data are expressed as values ± SEM of the ratio of green to red fluorescence signal intensity (ox/non-ox). (*n* = 3 experiments at least). Statistical test: unpaired *t*-test. **G** Schematic representation of glycolysis and lipolysis metabolism in ARPE-19 WT and ARPE-19^shREP1^ cells. Whereas in WT cells glucose uptake guarantees glycolysis activation and mitochondria function, in *sh*REP1 cells the block of glycolysis promotes lipolysis increase and mitochondria disruption. **H** Graphs show the decrements of oxygen consumption rates after the injections of BPTES, Etomoxir and UK5099 in ARPE-19 WT, ARPE-19^shREP1^ cells. The values are expressed as means ± SEM. Statistical analysis: unpaired *t*-test.

Given that the absence of glycolysis triggers a shift toward increased lipid degradation to sustain energy production [34], we investigated whether the lack of glucose uptake could influence lipid levels in ARPE-19^sh-REP1^ cells. Gene Ontology and Functional Annotation Clustering analyzes on transcriptomic data showed that expression pattern of genes associated with lipid metabolism was significantly increased (Fig. 4A, B) in ARPE-19^sh-REP1^ compared to control cells. Lipid droplets staining and imaging supported these results. Quantitative analyzes revealed that abundance and dimensions of intracellular lipid droplets are increased in ARPE-19^sh-REP1^ compared to control cells (Fig. 4C, D). Notably, when glycolysis is impaired, cells use alternative ways to generate energy, and one of the primary pathways they turn to is lipid oxidation to produce ATP in the absence of sufficient glucose availability. Next, we assessed whether lipid oxidation was affected in the absence of glucose uptake in ARPE-19^sh-REP1^ cells. In accordance with this hypothesis, bodipy staining assays revealed an increase of lipid oxidation in ARPE-19^si-REP1^ compared to control cells (Fig. 4E, F), suggesting a switch in metabolism of mitochondria from glucose to lipid oxidation (Fig. 4G). To get insight into the mechanism of mitochondrial metabolic switch in ARPE-19^sh-REP1^, we tested the oxidative capacity on different available mitochondrial substrate (glucose/pyruvate, glutamine/glutamate, long-chain fatty acids) and measured mitochondrial respiration in the presence of specific inhibitors, namely UK5099 for glucose/pyruvate, BPTES for glutamine/glutamate or Etomoxir for fatty acid beta-oxidation. As expected, we did not observe a reduction in oxygen consumption rate following the UK5099 administration on ARPE-19^sh-REP1^ compared to control cells (Fig. 4H), supporting the idea of defective uptake and mitochondrial use of glucose as a fuel in ARPE-19^sh-REP1^. Consistently, both BPTES and Etomoxir administration induced a reduction in oxygen consumption rate in ARPE-19^sh-REP1^, demonstrating that mitochondria consume oxygen to “burn” carbon intermediates derived from lipid as a fuel source to produce ATP (Fig. 4H).

### Depletion of REP-1 recapitulates the CHM phenotype in medaka fish

Next, we explored the consequences of depletion of Rep-1 in vivo. We carried out a loss-of-function analysis in the Medaka fish (*O. latipes*, Ol) model system using gene knockdown with a specific morpholino (MO) directed against the 5’UTR of medaka *olRep-1* ortholog present in the UCSC Genome Browser [(October 2005 v.1.0): ENSORLT00000016223.1 at chr14:24.047.874-24.056.452]. Depletion of *olRep-1* caused a delay in larvae hatching associated at stage (St) 40 with noticeably larger yolk sacs, reduced eye size accompanied by reduced of cone photoreceptor outer segment (OS) (Supplementary Fig. 2A–D) and led to a moderate embryonic lethality, in line with previous results [35]. To determine whether the *olRep-1* morphants phenotype was indeed related to abnormal mitochondrial metabolism, we analyzed glucose uptake and mitochondria structure. Consistent with our in vitro data, we observed decreased levels of mitochondrial Tomm20 and Cs proteins as well as a reduction of Glut1 and pAkt signaling (Supplementary Fig. 2E) associated with a deficit of glucose uptake in *olRep-1* morphants (Supplementary Fig. 2F). Notably, immunofluorescence analysis demonstrated that RPE cells of *olREP-1* morphants had disorganized and fragmented mitochondria (Supplementary Fig. 2G), associated with increased ROS production (Supplementary Fig. 2H). Mutations in *Rep-1* gene induce reduction of phospholipids including structural lipids [36]. Consistently with literature, lipidomics analysis demonstrated that *olRep-1* morphants had a decrease in phosphatidylcholines (PCs) PC 18:0/18:2, PC 16:0/22:6 and PC 18:1/22:6 (Supplementary Fig. 2I), which play a crucial role in mitochondrial integrity, function and energy production [37] and are key structural lipids of photoreceptor cell membranes, especially in the outer segments of rods and cones [36, 38].

### Pharmacological Leptin administration rescues glucose/lipid unbalance in vitro

**Fig. 5:**
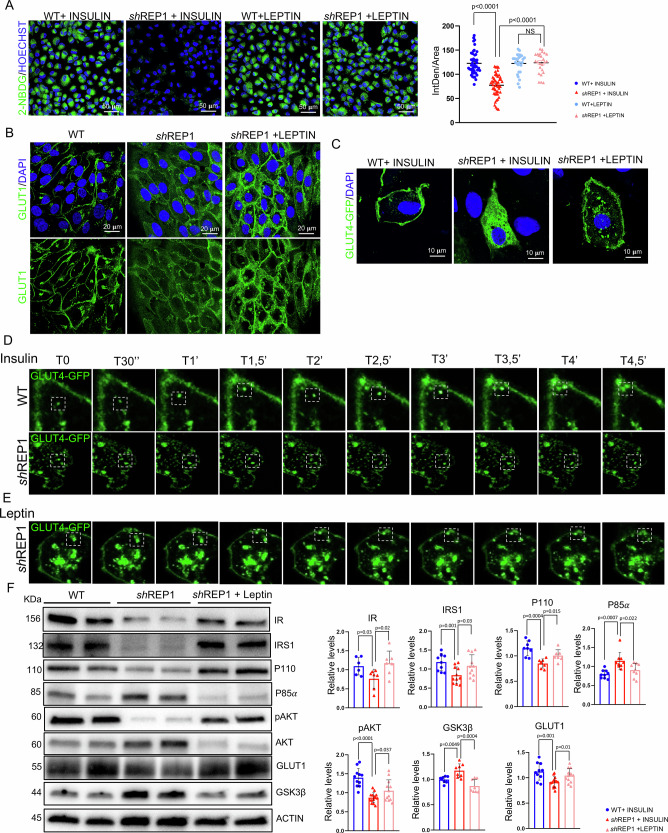
Pharmacological administration of leptin restores glucose/lipid balance in vitro. **A** Representative confocal image of 2-NBDG in ARPE-19 WT and ARPE-19^shREP1^ for glucose uptake evaluation after insulin (1 µM) and leptin (150 nM) stimulation. Nuclei were counterstained with HOECHST (blue). Scale bar 50 µm. The Graph on the right shows single values of the normalized fluorescence intensity (IntDen/Area) of 2-NBDG-positive cells ± SEM (n = 3 experiments at least). Statistical test: unpaired *t*-test. **B** Immunofluorescence labeling images of GLUT1 (green) and DAPI (blue) in ARPE-19 WT and ARPE-19^shREP1^ and leptin-treated ARPE-19^shREP1^. Scale bar 20 µm. **C** Representative immunofluorescence image of GLUT4-GFP translocation to the plasma membrane in insulin and leptin-stimulated ARPE-19 WT and ARPE-19^shREP1^. Cells were transfected with GFP-tagged GLUT4. After 24 h cells were stimulated with Insulin (1 µM; 30’) or leptin (150 nM; 30’), fixed and analyzed by confocal microscopy. Nuclei were counterstained with DAPI. Scale bar 10 µm. Live cell imaging of GLUT4-GFP (green) vesicles translocation from the cytoplasm to the plasma membrane in ARPE-19 WT and ARPE-19^shREP1^ cells without insulin stimulation (T0) and with a progressive insulin stimulation (from T30” to T4,5’), (**D**) and without leptin (T0) and with a progressive leptin stimulation (**E**). White boxes are magnifications showing GLUT4 migration. Scale bar 1 µm. **F** Immunoblots and calculated levels (right) of insulin receptor (IR), insulin receptor substrate1 (IRS1) and some members of its signal transduction network PI3K p110, PI3K p85α, pAKT (ser473), AKT, GLUT1, and GSK3β evaluated in ARPE-19 WT and ARPE-19^shREP1^ and Leptin stimulated-^shREP1^. Data are expressed as values of IR, IRS1, PI3K p110, PI3K p85, pAKT (ser473)/ AKT ratio, GLUT1, and GSK3β ± SEM (*n* = 3 experiments at least). Statistical test: unpaired *t*-test.

Impairment of the glucose uptake has been frequently implicated in the glucotoxicity-mediated retinal dystrophy, which is worsened by the dysfunctional of lipid metabolism and mtROS production [39, 40]. Recent studies have shown that the satiety-regulating hormone leptin may result as a potential therapeutic drug for inducing glucose uptake through Glut4 translocation to the plasma membrane of neuronal cells. We hypothesized that a leptin-mediated induction of GLUT4 translocation should re-establish normal both glucose uptake and mitochondrial metabolism and rescue retinal phenotypes both in vitro and in vivo, respectively. Notably, culturing ARPE-19^sh-REP1^ cells in the presence of Leptin led to a significant increase in glucose uptake associated with the translocation of both GLUT1 and GLUT4 to the plasma membrane (Fig. 5A–E and Movie S3). Consistently, we noted that administration of Leptin increased the expression of IR and IRS1 accompanied by rescue of GSK3-β expression level and activation of PI3K/AKT pathways as demonstrated by western blot analysis for p110, p85α, and pAKT/AKT markers (Fig. 5F), supporting the notion that pharmacological treatment restores glucose metabolism in ARPE-19^sh-REP1^ cells treated with Leptin compared with vehicle control cells. Importantly, Leptin also alleviates lipotoxicity by promoting lipid oxidation and reducing lipid accumulation in cells where excessive lipids alter cellular homeostasis and function [41]. We next asked whether the Leptin administration may equally induce possible changes in lipid metabolism, which was affected in ARPE-19^sh-REP1^ cells due to lack of glucose uptake. Consistent with the above data, the treatment with Leptin in in ARPE-19^sh-REP1^ cells was sufficient to restore lipid accumulation (Supplementary Fig. 3A, B) and accordingly, this effect was associated with an increase of lipid oxidation and transcriptome rewiring due to the induction of lipids metabolism as shown in Gene Ontology and Functional Annotation Clustering analyzes (Supplementary Fig. 3C–F).

### Leptin re-established mitochondrial homeostasis and restored visual loss in CHM medaka model

**Fig. 6:**
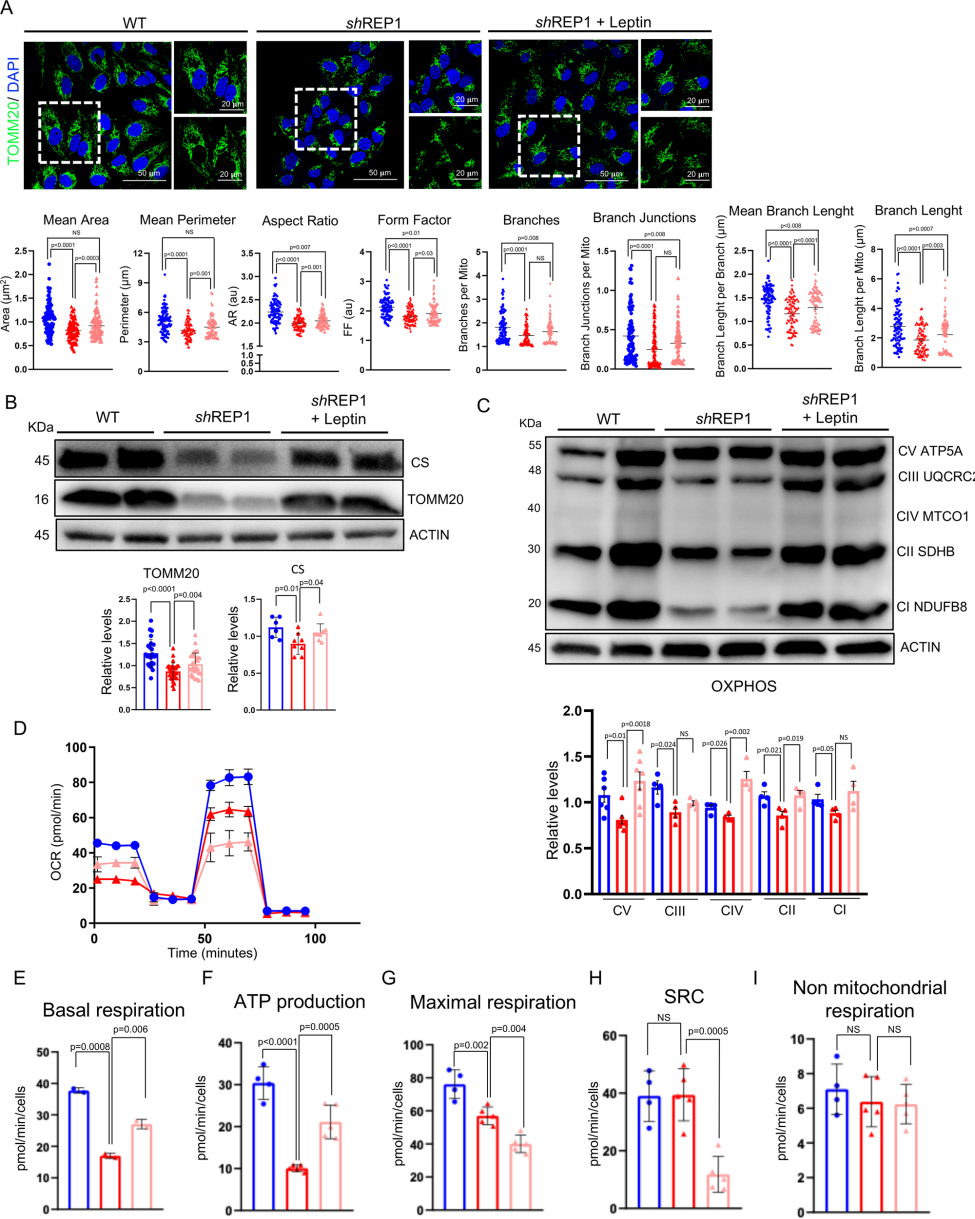
Leptin re-established normal mitochondrial function in vitro. **A** Representative confocal images of TOMM20-labeled mitochondria (green) in ARPE-19 WT, ARPE-19^shREP1^ and leptin-treated- ARPE-19^shREP1^. Nuclei were counterstained with DAPI (blue). Scale bar 50 µm. Magnified box: scale bar 20 µm. Graphs show the quantitative comparison of the mitochondrial morphology and network connectivity parameters between ARPE-19 WT, ARPE-19^shREP1^ and Leptin-treated ARPE-19^shREP1^. All data are displayed as values ± SEM. Statistical analysis: unpaired *t*-test. **B** Representatives immunoblot images and calculated levels (down) of TOMM20 and Citrate synthase in ARPE-19 WT, ARPE-19^shREP1^ and Leptin-treated shREP1. Data are expressed as mean ± SEM of the levels of TOMM20 and Citrate synthase relative to β-Actin, used as loading control. (*n* = 3 experiments at least). Statistical test: unpaired *t*-test. **C** Representative Western blot analysis of OXPHOS mitochondrial subunits performed with an antibody cocktail specific for representing the five mitochondrial oxidative phosphorylation complexes. The histogram represents the values ± SEM of the ATP5A, UQCRC2, SDHB, MTCO1 and NDUFB8 protein expressions levels relative to β-Actin, used as loading control (*n* = 3 experiments). Statistical test: unpaired *t*-test. **D**–**I** Representative graphs of Cell Mito Stress assay performed by Seahorse XF analyzer in ARPE-19 WT, ARPE-19^shREP1^ cells, and after treatment with Leptin (150 nM for 3 h). In the bar charts (**D**), each point in the OCR time courses is the average of three technical replicates. Basal respiration (**E**), ATP production (**F**), maximal respiration (**G**), spare respiratory capacity (**H**) and non-mitochondrial respiration (**I**) are reported in the histograms. The values are expressed as means ± SEM. Statistical analysis: unpaired *t*-test.

Restoring glucose uptake and lipid metabolism induces rescue of mitochondrial structures and metabolism in conditions like diabetes [42]. We therefore verified the impact of glucose uptake on mitochondrial dynamics and function. Figure 6 and Supplementary Fig. 4 show that Leptin stimulation rescued mitochondrial structure and shape as shown by measurement for area, perimeter, FF, AR and branching parameters (Fig. 6A and Supplementary Fig. 4A, B), supporting a rescue in overall connectivity and morphological complexity. We confirmed this observation by quantifying both TOMM20 and CS immunostaining (Fig. 6A, B and Supplementary Fig. 4A, B). Notably, these changes were also accompanied by a significant restoration of OXPHOS protein expression in Leptin-treated ARPE-19^sh-REP1^ compared to control cells (Fig. 6C). These findings were confirmed by measuring complex activities on isolated mitochondria of Leptin-treated ARPE-19^sh-REP1^ compared to control cells. Notably, mitochondrial complexes I-II and complex IV activities were fully repristinated (Supplementary Fig. 4C). Consistent with this observation, the oxygen consumption rates were also measured in WT, ARPE-19^sh-REP1^, and ARPE-19^sh-REP1^ cells after treatment with Leptin. Notably, we observed a significant recovery of OCR levels to basal state (Fig. 6D, E) and in ATP production (Fig. 6F) in ARPE-19^sh-REP1^-treated cells when compared to ARPE-19^sh-REP1^ cells. However, maximal respiratory capacity was reduced in ARPE-19^sh-REP1^ cells compared to WT cells, and further reduced following treatment, probably indicating maximum ATP synthesis efficiency in the latter (Fig. 6G). In fact, following treatment, ARPE-19^sh-REP1^ cells show the lowest levels of respiratory reserve capacity (Fig. 6H), indicating their inability to eventually increase oxidative capacity to support additional energetic demands. No difference was found in non-mitochondrial respiration (Fig. 6I). In order to test the oxidative capacity on the different mitochondrial fuel available (glucose/pyruvate, glutamine/glutamate, long-chain fatty acids), we also measured mitochondrial respiration in the presence of fuel pathway inhibitors: UK5099, BPTES and Etomoxir. Notably, leptin treatment induced a significant reduction of the oxidative capacity in ARPE-19^sh-REP1^-treated cells following acute injection of UK, comparable to the same decrease observed in WT cells. This finding supported the recovery of the glucose oxidation pathway in treated cells compared to ARPE-19^sh-REP1^ cells (Supplementary Fig. 4D). In accordance, measurements of both H_2_DCFDA and Mitosox staining were also restored in Leptin-treated ARPE-19^sh-REP1^ compared to control cells (Supplementary Fig. 5A, B), supporting a functional restoration of mitochondria. Concordantly, the above-described mitophagy phenotype associated with the selective clearance of damaged mitochondria in ARPE-19^sh-REP1^ cells was significantly restored when the ARPE-19^sh-REP1^ cells were treated with Leptin (Supplementary Fig. 5C).

**Fig. 7:**
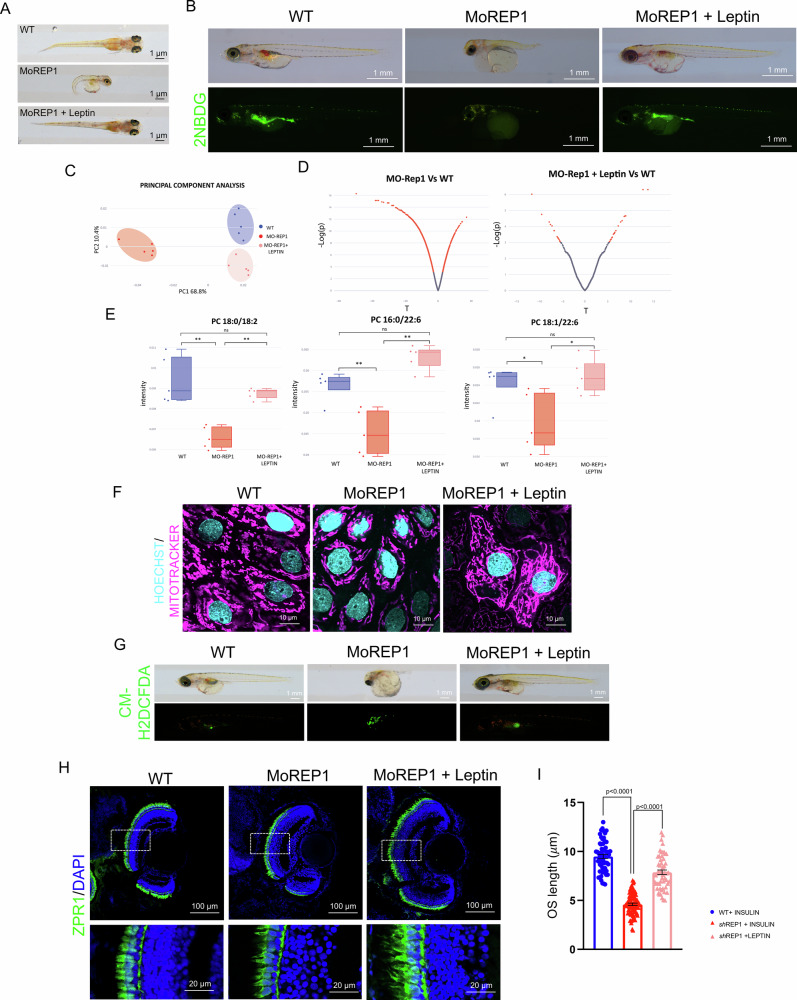
Leptin improved visual function in the CHM medaka model. **A** Stereo-microscopic representative images of WT, MoREP-1 and MoREP-1 leptin-treated medaka at stage 40. Scale bar 1 mm. **B** Representative images of 2-NBDG (0.6 mM; 4 h), a fluorescent analogue of glucose, for evaluation of glucose uptake in WT, MoREP-1 and after leptin treatment in Morphants medaka at stage 40. Scale bar 1 mm. **C** Principal component analysis (PCA) of ESI + MS data showing distinct clustering of WT (green), diseased (red), and leptin-treated (blue) medaka, indicating restoration of the lipidomic profile upon therapy. **D** The left panel shows the comparison between MO-Rep1 and WT, while the right panel compares leptin-treated MO-Rep1 to WT. Each dot in the two-sided plot represents a metabolite, plotted by its *t*-statistic on the *x*-axis and the –log₁₀(*p*-value) on the *y*-axis. Orange dots indicate metabolites showing statistically significant differences between the two groups (FDR-adjusted *p* < 0.05). **E** Quantitative analysis of phosphatidylcholine (PC18:0/18:2, PC16:0/22:6, PC18:1/22:6) species identified as biomarkers of choroideremia. One-way ANOVA test revealed significative changes (*p*-corrected values < 0.05); therefore, Tukey’s test was used to assess pairwise differences. (^**^p < 0.01, ^*^0.01<*p* < 0.05; ns = not significant). The analysis reveals significant reduction in diseased medaka compared to WT, with partial or complete restoration following leptin treatment. (*n* = 5 per group); The error bars indicate the interquartile range (IQR). **F** Representative confocal live images of RPE mitochondria stained in live with Mitotracker Deep Red. Nuclei were counterstained with HOECHST. Scale bar: 10 µm. **G** Fluorescence images of live detections of intracellular ROS in stage 40 medaka WT, MoREP1 and MoREP1 leptin treated, using the fluorescent probe CM-H2DCDFDA. **H** Immunofluorescence labelling images of ZPR1 in WT, MoREP-1 and MoREP-1 leptin-treated fish. Scale bar 100 µm. Magnified views of the regions in the boxes are provided in the bottom. Scale bar 20 µm. **I** The graph shows the outer segment length (μm) quantification in WT, MoREP1 and MoREP1 leptin-treated stage 40 medaka fish. The histogram displays single values and data are expressed as mean ± SEM of at least three independent experiments. Student’s *t* test.

Based on the above findings, we figured that if Leptin directly ameliorates the mitochondrial structure and function through an increase of glucose uptake and restoration of lipid metabolism, the pharmacological administration of Leptin to MoRep-1 morphant embryos should prevent the CHM phenotype in Medaka fish. Notably, daily treatment of MoRep-1 morphants with Leptin, from St.30 onward, was sufficient to restore phenotype, glucose uptake and lipids profile. Lipid profiling revealed marked alterations in lipid composition between wild-type (WT) and diseased fish. A pairwise comparison of lipidic profiles revealed numerous significantly altered metabolites in the MO-Rep1 group compared to WT, as shown by *t*-tests. In contrast, the number of significant alterations was markedly reduced in the CHM phenotype of medaka fish after treatment with leptin (Fig. 7A–E, Supplementary Fig. 6A–C and Table S3). Principal component analysis (PCA) of the ESI+MS dataset further substantiated these findings, demonstrating a clear separation between WT and MO-Rep1 groups, thereby confirming the presence of a consistent, disease-specific lipidomic signature. Strikingly, the lipid profiles of leptin-treated medaka closely aligned with those of WT fish, suggesting a robust therapeutic effect and restoration of lipid homeostasis. Moreover, exposure of MoRep-1 morphant embryos to leptin rescued the mitochondrial structure and oxidative stress (Fig. 7F, G) due to mtROS, strongly supporting a full recovery of mitochondrial function. Importantly, we observed a significant increase in the number of preserved PR cones at the ONL and in the Leptin-treated eyes compared to controls (Fig. 7H). At this stage, we also detected an increase of OS length for cone PR markers (Fig. 7I). Altogether, these data shed light on the impact of the consequences of REP-1 gene loss on glucose/lipid metabolism, as well as its broader impact on mitochondrial function. In this context, it would be relevant to determine whether the proposed metabolic dysfunction for the retina in choroideremia is merely an extreme manifestation of a systemic defect, potentially leading to additional metabolic complications. Here, we propose that Leptin administration could serve as a promising therapeutic approach to restore lipid metabolism and mitochondrial function by enhancing glucose uptake. Further research is necessary to assess the clinical significance of these results, with the ultimate goal of mitigating pathological conditions in patients affected by Choroideremia.

## Discussion

Glucose is the primary metabolic source of carbon for ATP production by mitochondria essential for neuronal bioenergetics. Retina metabolism relies heavily on glucose to sustain photoreceptor activity, neurotransmission, and cellular homeostasis. Glucose is metabolized through glycolysis, oxidative phosphorylation, and the pentose phosphate pathway to generate ATP by mitochondria [43]. Consequently, mitochondrial dynamics must undergo adaptive modulation in response to change in glucose availability, ensuring efficient ATP generation during the vision. Beyond energy production, glucose metabolism is intricately linked to retinal angiogenesis and vascular homeostasis [44]. Disruptions in glucose uptake can contribute to pathological conditions such as diabetic retinopathy, where aberrant glucose metabolism leads to oxidative stress, inflammation, and vascular dysfunction [45]. Therefore, how altered glucose uptake may influence both physiological and pathological processes represent key questions that have been poorly addressed. Here, we identify a novel regulatory mechanism essential for GLUT4 trafficking to plasma membrane mediated by REP-1. In particular, by developing a RPE cell-based Choroideremia disease model, we show that REP-1 is necessary for GLUT4 plasma membrane translocation, which is required for ensuring physiological glucose uptake. REP-1 LoF caused an increase in GLUT4-vesicle retention at cytoplasm, affecting glucose uptake and glycolysis pathway and consequently the mitochondria respiratory chain associated with mtROS production. Our data clearly show a metabolic switch from glycolysis to lipid oxidation at transcriptional and functional levels, regulated by the loss of REP-1, demonstrating that further insulin-regulated signaling network downstream glucose uptake is deficient in REP-1-depleted cells and in negative feedback could participate in pathological phenotype. Consistent with this hypothesis, peripheral blood analyzes in CHM patients revealed crystalline inclusions within lymphocytes, alterations in plasma lipid composition, and structural abnormalities of erythrocyte membranes, collectively associated with an increased incidence of systemic metabolic disorders, most notably diabetes, hypercholesterolemia, and hyperglycemia [46], suggesting that the ocular phenotype represents the most clinically apparent manifestation of Choroideremia, but at same time it is embedded within a broader metabolic syndrome. Recognition of this correlation reframes CHM as a multisystem disorder, highlighting glucose and lipid metabolism as a potential therapeutic axis. It is well-studied and accepted phenomenon that local hyperglycemia is associated with glucotoxicity leading to tissue and cell dysfunction [47], while mitochondrial energetic metabolism shifts toward other sources of energy such as lipids [48]. Notably, while the main energy source is shifted to lipids, lipogenesis and lipid oxidation are both induced and chronically activated to produce ATP and NADH to support cell metabolism. We also show that REP-1 LoF was mirrored by an increase of transcriptional regulation of lipids and cholesterol-associated genes, whose expression could increase the lipogenesis and cholesterol biosynthesis. In this context, we found that REP-1-depleted cells accumulated lipid droplets. Notably, hypercholesterolemia and significant fatty acid abnormalities were also associated in CHM families [46, 49]. Thus, it would be relevant to determine whether our proposed metabolic dysfunction for choroideremia is purely an extreme manifestation of a systemic pathology, leading to additional metabolic complications. In this context, recently, the concept that common retinal diseases that were assumed to be restricted to the retina turned out to be systemic diseases after careful phenotyping is gaining [50]. Importantly, unlike other cells, neurons do not rely on lipids for secondary sources of energy, but they employ lipids for structural components of their membranes or signaling molecules [51]. This is largely due to the low expression of enzymes necessary for fatty acid β-oxidation in neuronal mitochondria, as well as the potential for lipid metabolism to generate ROS, which can be detrimental to neuronal function. Thus, lipids are accumulated and stored as lipid droplets. Recent findings point out the lipid metabolic cycle between neurons and glia, which contributes to the regulation of neuronal energy homeostasis by metabolizing excessive fatty acids and lipids, providing neurons with alternative substrates such as ketone bodies or lactate, which neurons can use for oxidative phosphorylation [52]. However, glucose deprivation due to absence of GLUTs may severely alter the ability of glial cells to support neurons by consuming lipid [53]. Furthermore, prolonged or excessive local hyperglycemia can have profound effects on glial cell function, leading to metabolic disturbances, oxidative stress, and neuroinflammation, which finally exacerbates degeneration [54]. Therefore, our findings support the notion that a chronic imbalance in mitochondria metabolism driven by lack of glucose uptake is not completely compensated by lipid oxidation, guiding an accumulation of lipids or products of reprogramming of lipid metabolism, which may lead to cellular lipotoxicity. Notably, several studies reported that restoration of glucose metabolism and lipotoxicity in insulin-deficient mice by leptin administration [55, 56]. Leptin binding to its long-form receptor (Ob‑Rb) may activate JAK2, which in turn recruits and stimulates PI3K. The generation of PIP3 by PI3K facilitates the recruitment and phosphorylation of Akt, a central metabolic regulator [57]. Activated Akt phosphorylates AS160 (Akt substrate of 160 kDa), a Rab GTPase-activating protein that normally restrains GLUT4 storage vesicles in the cytoplasm [57]. Phosphorylation of AS160 inhibits its GAP activity, thereby allowing Rab proteins to remain in their GTP-bound active state. This switch promotes GLUT4-enriched vesicle trafficking toward the plasma membrane [57]. In parallel, leptin signaling intersects with AMPK and PKC cascades, which remodel the cytoskeleton and promote vesicle docking and fusion. The integrated outcome of these pathways is an increased density of GLUT4 transporters at the cell surface, enhancing glucose uptake [58]. While insulin and leptin share overlapping intermediates, leptin’s action is particularly relevant in neuronal cells, where it augments glucose utilization independently of insulin, linking energy homeostasis with nutrient uptake [57]. Importantly, Leptin also emerged as a pivotal regulator of mitochondrial metabolism, exerting direct effects on fatty acid oxidation and organelle homeostasis. Leptin-mediated activation of AMPK may in turn promote the expression and activity of carnitine palmitoyltransferase-1 (CPT-1), the rate-limiting enzyme for mitochondrial fatty acid entry [59]. This activation enhances β-oxidation, thereby increasing ATP production and reducing lipid accumulation. In parallel, leptin upregulates peroxisome proliferator-activated receptor γ coactivator-1α (PGC-1α) [60], a master regulator of mitochondrial biogenesis, and modulates uncoupling proteins (UCPs) to optimize respiratory efficiency. These coordinated actions not only stimulate substrate oxidation but also preserve mitochondrial morphology, reduce oxidative stress, and maintain redox balance [59].In this perspective, it would be important to establish whether the proposed therapeutic regime for CHM, which is based on inducing glucose uptake and increasing lipid oxidation and consumption, may counteract the onset and progression of CHM disease. Here, we propose that pharmacological Leptin administration represents an innovative therapeutic approach to restore mitochondrial structure and function by reactivating glycolysis. Additional investigations are necessary to assess the clinical significance of our findings, aiming to mitigate retinal pathological conditions in CHM patients.

## Supplementary information

SUPPLEMENTARY INFORMATION

Supplementary Figure 1

Supplementary Figure 2

Supplementary Figure 3

Supplementary Figure 4

Supplementary Figure 5

Supplementary Figure 6

Supplementary Table 1

Supplementary Table 2

Supplementary Table 3

Uncropped WB Data

Supplementary movie 1

Supplementary movie 2

Supplementary movie 3

## Data Availability

All datasets generated or analyzed during this study are included in the published article. Any additional information required to reanalyze the data reported in this paper is available from the lead contact upon request.

## Code availability

This study generated GEO Submission number (GSE299462).
