# Supplementary material for: REP-1 deficiency induces aberrant mitochondrial metabolic rewiring from glycolysis to lipid oxidation in CHM disease
Source: Cell Death Dis. 2026 Mar 30;17(1):436. doi: 10.1038/s41419-026-08592-6 (PMC13158304; doi:10.1038/s41419-026-08592-6)
Supplement: Supplementary file 1 — SUPPLEMENTARY INFORMATION [file 41419_2026_8592_MOESM1_ESM.docx]

**SUPPLEMENTARY INFORMATION**

**Supplementary Fig.1. REP-1 deficiency drives alteration in mitochondrial morphology and function**. **A)** Representative western blot analysis of REP1 performed in ARPE-19 WT and ARPE-19^shREP1^ cell lysates. Graph on the right shows the values ± SEM of the REP1 protein expression levels relative to β-Actin, used as loading control. (n=3 experiments at least). Statistical test: unpaired t-test. **B)** Representative histogram of REP-1 expression levels by quantitative RT-PCR analysis in ARPE-19^shREP1^ cells. Results are shown as the fold change ± SEM of the REP1 levels relative to one of the WT samples, used as reference (n=3 experiments at least). Statistical test: unpaired t-test. **C-F)** Representative confocal images of Citrate Syntase- (green) **(C)** and MitoTracker Deep Red- (magenta) **(E)** labeled mitochondria in ARPE-19 WT and ARPE-19^shREP1^. Nuclei were counterstained with DAPI or HOECHST. Graphs **(D, F)** show the quantitative comparison of the mitochondrial morphology and network connectivity parameters between ARPE-19 WT and ARPE-19^shREP1^. All data are represented as values ± SEM of at least 3 independent experiments. Statistical analysis: unpaired t-test. **G)** Mitochondrial respiratory complexes activities detected in isolated mitochondria from ARPE-19 WT and ARPE-19^shREP1^ cells. Data are represented in the graphs as means ± SEM of six determinations from different cells extracts. Each determination is the mean of three independent measures. Statistical analysis: unpaired t-test. **H)** Western blot analysis of LC3-I and LC3-II. Cells were grown under normal (CTRL) or starvation (STV) conditions in the presence or absence of autophagy inhibitor bafilomycin A1 (baf, 200 nM) for 3h. Right panel shows quantitative analysis of LC3-II protein expression levels. The mean ± SEM of values for protein bands, each normalized on the relative values of Actin, are reported in the histograms. Statistical test: Student’s t-test.

**Supplementary Fig.2. Loss of REP-1 mirrors the CHM phenotype in medaka fish.** **A)** Representative western blot image and calculated levels (right) of REP-1 in stage 24 medaka WT and MoREP1 lysates. Data are expressed as values ± SEM of the REP1 levels relative to β-Actin, used as loading control. (n=3 experiments at least). Statistical test: unpaired t-test. **B)** Stereo-microscopic representative images of WT and MoREP-1 medaka at stage 40. Scale bar 1 mm. **C-D)** Immunofluorescence labelling images of Zpr1 **(C)** and Rho **(D)** in WT and MoREP-1 stage 40 fish. Scale bar 100µm. Magnified views of the regions in the boxes are provided at the bottom. Scale bar 20µm. **E)** Demonstratives immunoblot images of Tomm20, Citrate synthetase, Glut1, pAkt(ser473) and Akt in stage 24 medaka WT and MoREP1 lysates. Histograms on the right represents the values ± SEM. β-Actin was used as loading control (n=3 experiments at least). Statistical test: unpaired t-test. **F)** Representative images of 2-NBDG uptake in WT and MoREP-1 medaka at stage 40. Experimental replicates: 3 at least. **G)** Confocal live images of RPE mitochondria stained in live with Mitotracker in WT and MoREP-1 medaka at stage 40. Nuclei are stained with HOECHST (n=3 experiments at least). Scale bar: 100 µm. **H)** Fluorescence images of representative detections of reactive oxygen species in stage 40 medaka WT and MoREP1, using the fluorescent probe CM-H2DCDFDA. **I)** The box plots compare the levels of three phosphatidyl cholines (PCs) among the different groups. B) PC 18:0/18:2, C) PC 16:0/22:6, and D) PC 18:1/22:6. Statistical analysis unpaired t-test. The error bars indicate the interquartile range (IQR).

**Supplementary Fig.3. Leptin restores lipid accumulation in ARPE-19 cells. A)** Representative confocal images of lipid droplets in ARPE-19 WT, ARPE-19^shREP1^ and leptin-treated ARPE-19^shREP1^ cells analyzed using Lipid Spot (green). Nuclei were counterstained with HOECHST (blue). Scale bar: 50µm. Magnified box scale bar 10 µm. **B)** Quantification of Lipid Droplets**.** Data are expressed as values ± SEM of the ratio of cell positive for lipid spot to HOECHST cells. (n=3 experiments at least). Statistical test: unpaired t-test. **C)** Representative confocal images of Lipid peroxidation evaluated by using BODIPY C11 fluorescence probe in ARPE-19 WT, ARPE-19^shREP1^ and leptin-treated ARPE-19^shREP1^ cells. Nuclei were stained with HOECHST. Scale bar 20µm. **D)** Fluorescence quantification of lipid peroxidation. Data are expressed as values ± SEM of the ratio of green to red fluorescence signal intensity (ox/non-ox). n=3 experiments at least. Statistical test: unpaired t-test. **E)** Heat map representing the expression of genes associated to lipid metabolism in Arpe-19 wild type cells, and upon shREP1 or Leptin treatment. **F)** Ballon plot representing the functional annotation analysis of the up-regulated genes in Arpe-19sh-REP1 upon Leptin treatment compared to control. Size and color indicate the Enrichment (−log10 of pvalue).

**Supplementary Fig.4. Leptin rescues mitochondrial morphology. A)** Representative confocal images of Citrate Synthase-labeled mitochondria (green) in ARPE-19 WT, ARPE-19^shREP1^ and leptin treated- ARPE-19^shREP1^. Nuclei were counterstained with DAPI. Lower Graphs show the quantitative comparison of the mitochondrial morphology and network connectivity parameters between ARPE-19 WT, ARPE-19^shREP1^ and Leptin-treated ^shREP1^. All data are represented as values ± SEM of at least three independent experiments. Statistical analysis: unpaired t-test. **B)** Representative confocal 3D-images of mitochondrial morphology from TOMM2 staining on ARPE-19 WT, ARPE-19^shREP1^ and leptin treated- ARPE-19^shREP1^ cells. **C)** Mitochondrial respiratory complexes activities detected in isolated mitochondria from ARPE-19 WT, ARPE-19^shREP1^ and leptin treated- ARPE-19^shREP1^ cells. In histograms the data were expressed as means ± SEM of three determinations from different cells extracts. Each determination is the mean of three independent measurements. Statistical analysis: unpaired t-test. **D)** Acute Response: in the bar charts are reported the decrements of oxygen consumption rates after the injections of BPTES, Etomoxir and UK5099 in ARPE-19 WT, ARPE-19^shREP1^ and leptin treated- ARPE-19^shREP1^. The values are expressed as means ± SEM. Statistical analysis: unpaired t-test.

**Supplementary Fig.5. Leptin affects ROS accumulation. A)** Confocal images of a live staining detection of intracellular ROS in ARPE-19 WT, ARPE-19^shREP1^ and leptin treated- ARPE-19^shREP1^, obtained by using the fluorescent probe CM-H2DCFDA. Nuclei were counterstained with HOECHST (blue). Scale bar 50µm. Data are expressed as values of fluorescence intensity ± SEM (n=3 experiments at least). Statistical test: unpaired t-test. **B)** Representative images of mitochondrial ROS using the fluorescent mitochondrial superoxide indicator MitoSOX, in ARPE-19 WT, ARPE-19^shREP1^ and leptin treated- ARPE-19^shREP1^cells. Nuclei were counterstained with HOECHST (blue). Scale bar 50µm. Magnified box scale bar 20µm. Data are showed as single values of the normalized fluorescence intensity (IntDen/Area) ± SEM (n=3 experiments at least). Statistical test: unpaired t-test. C**)** Immunofluorescence labelling images of TOMM20 (green), LAMP1 (red) and DAPI (blue) after 3h of BAF treatment in ARPE-19 WT (right), ARPE-19^shREP1^ (center) and leptin-treated ARPE-19^shREP1^(left). Scale bar 10µm. Magnified views of the regions in the boxes are provided in both Airyscan high-resolution microscopy and 3D-confocal microscopy (Scale bar: 2µm).

**Supplementary Fig.6.** Diagnostic fragments from MS/MS spectra of [M+Na]+ of **A)** PC 18:0 18:2, **B)** PC 16:0 22:6, and **C)** PC 18:1 22:6.

**Supplementary Table 1.** Excell file contains common DEGs of gene expression profiles from 3T3-L1^RAB10-KO^ and ARPE-19^sh-REP1^ cells.

**Supplementary Table 2.** Excell file contains DAVID gene ontology from 3T3-L1^RAB10-KO^ and ARPE-19^sh-REP1^ cells.

**Supplementary Table 3.** Excell file contains key lipids structure annotation. The multistep annotation was carried out via MZmine4, GNPS2 library matching and finally confirmed through manual spectra curation.

**Supplementary movie 1.** Insulin-mediated translocation of GLUT4 in WT ARPE-19 cells. Related to Fig. 5D.

**Supplementary movie 2.** Insulin-mediated translocation of GLUT4 in *sh*REP1 ARPE-19 cells. Related to Fig. 5D.

**Supplementary movie 3.** Leptin-mediated translocation of GLUT4 in *sh*REP1 ARPE-19 cells. Related to Fig. 5E.
