# Supplementary figures and images for: REP-1 deficiency induces aberrant mitochondrial metabolic rewiring from glycolysis to lipid oxidation in CHM disease
Source: Cell Death Dis. 2026 Mar 30;17(1):436. doi: 10.1038/s41419-026-08592-6 (PMC13158304; doi:10.1038/s41419-026-08592-6)

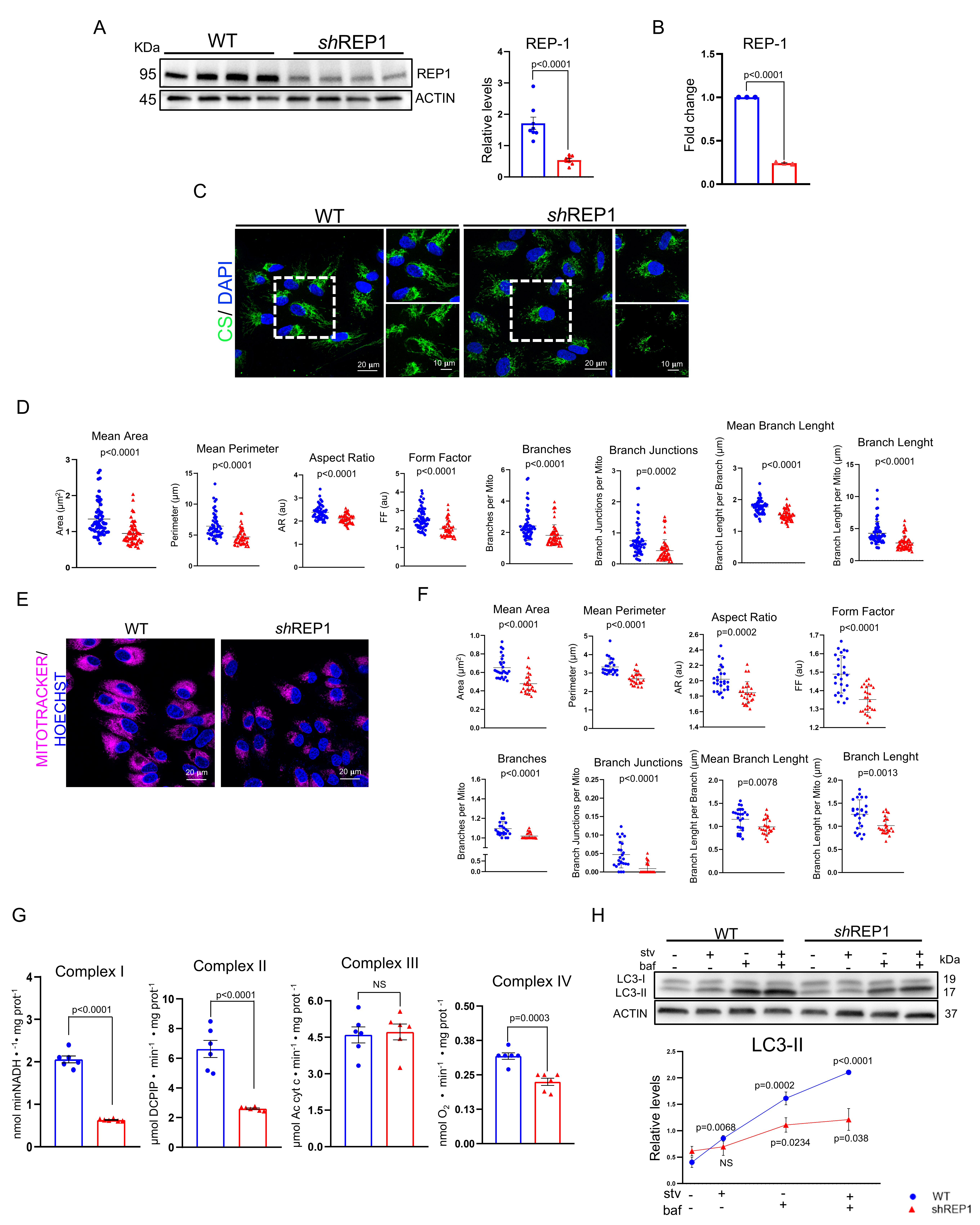

Supplement: Supplementary file 2 — Supplementary Figure 1 [file 41419_2026_8592_MOESM2_ESM.tif]

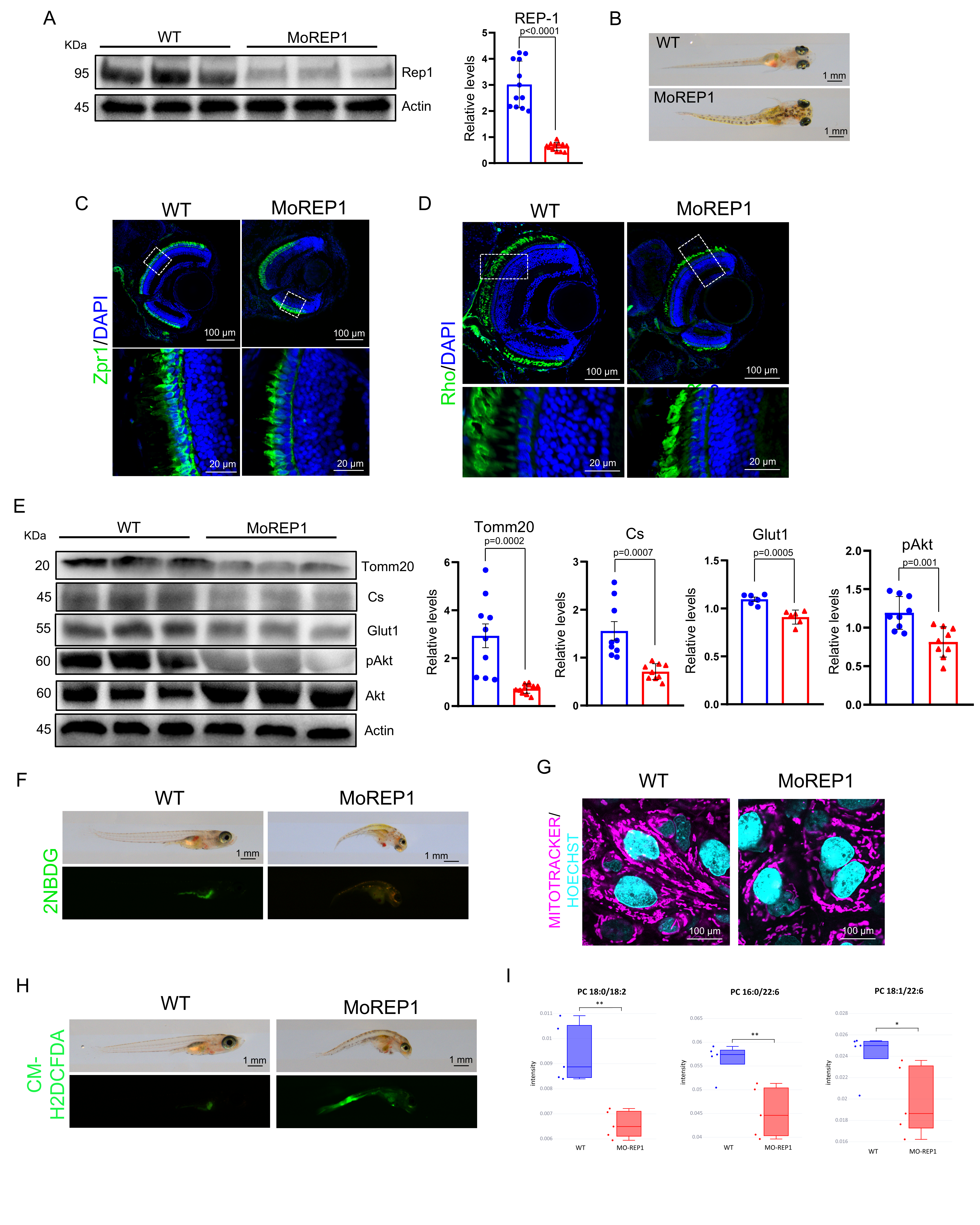

Supplement: Supplementary file 3 — Supplementary Figure 2 [file 41419_2026_8592_MOESM3_ESM.tif]

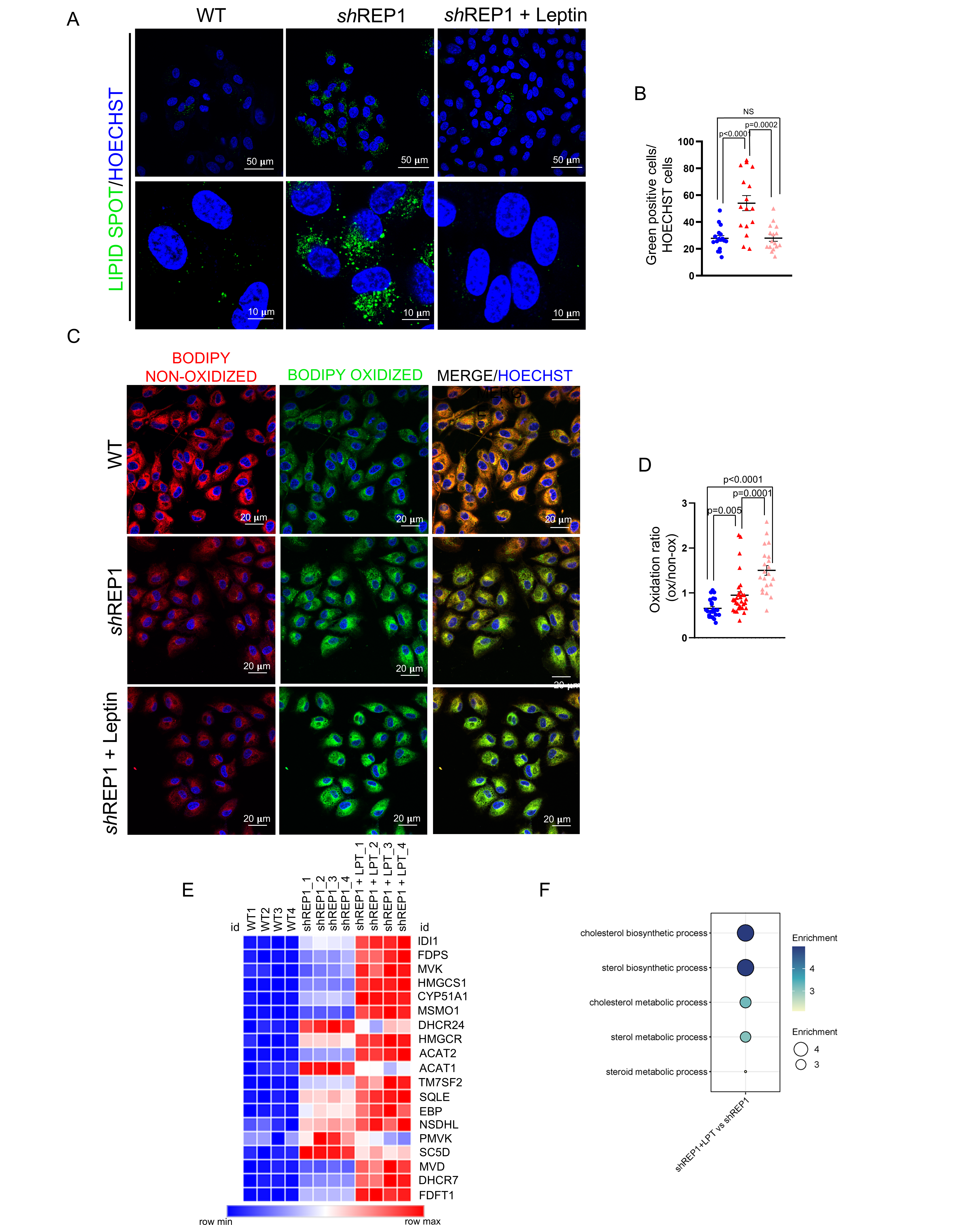

Supplement: Supplementary file 4 — Supplementary Figure 3 [file 41419_2026_8592_MOESM4_ESM.tif]

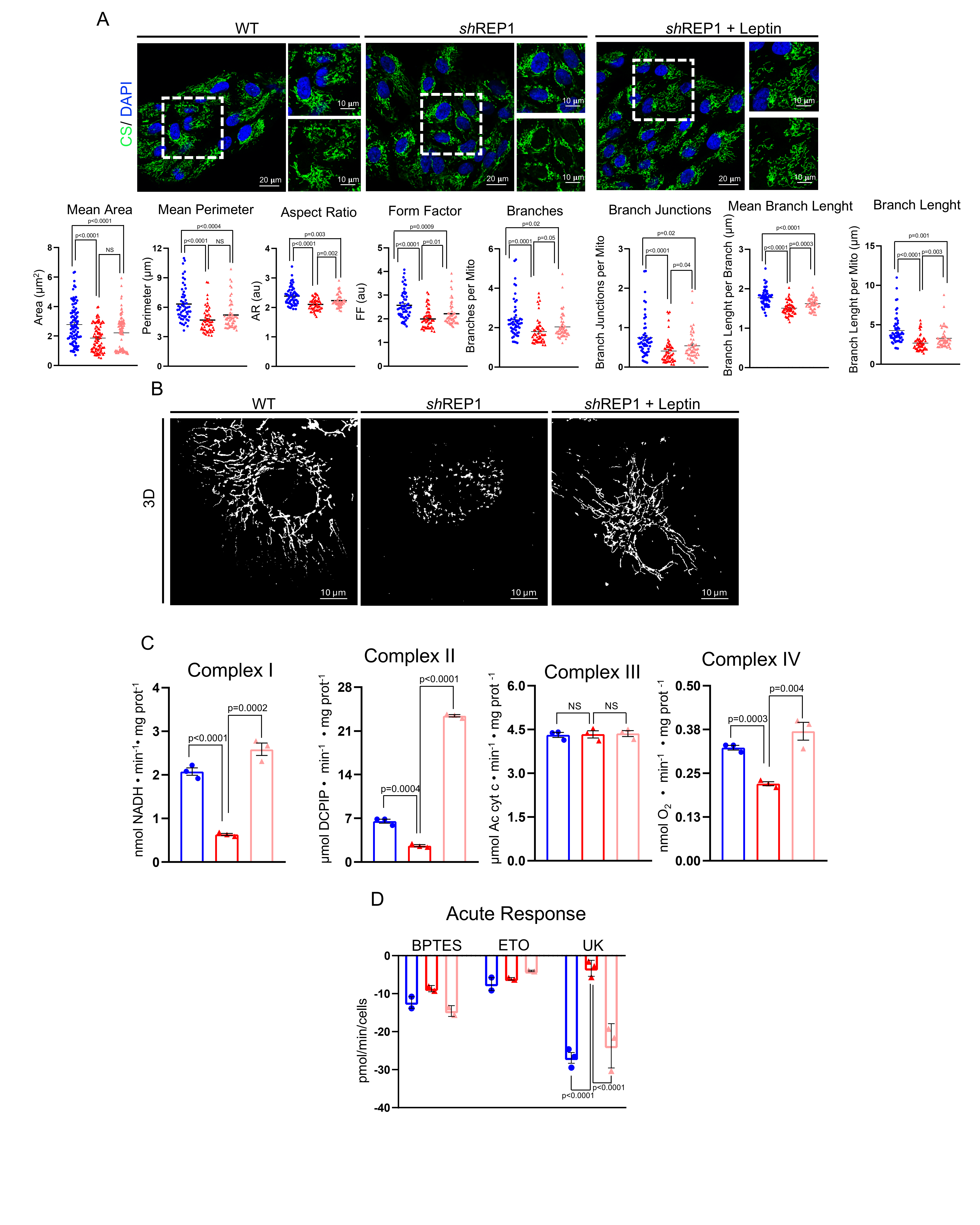

Supplement: Supplementary file 5 — Supplementary Figure 4 [file 41419_2026_8592_MOESM5_ESM.tif]

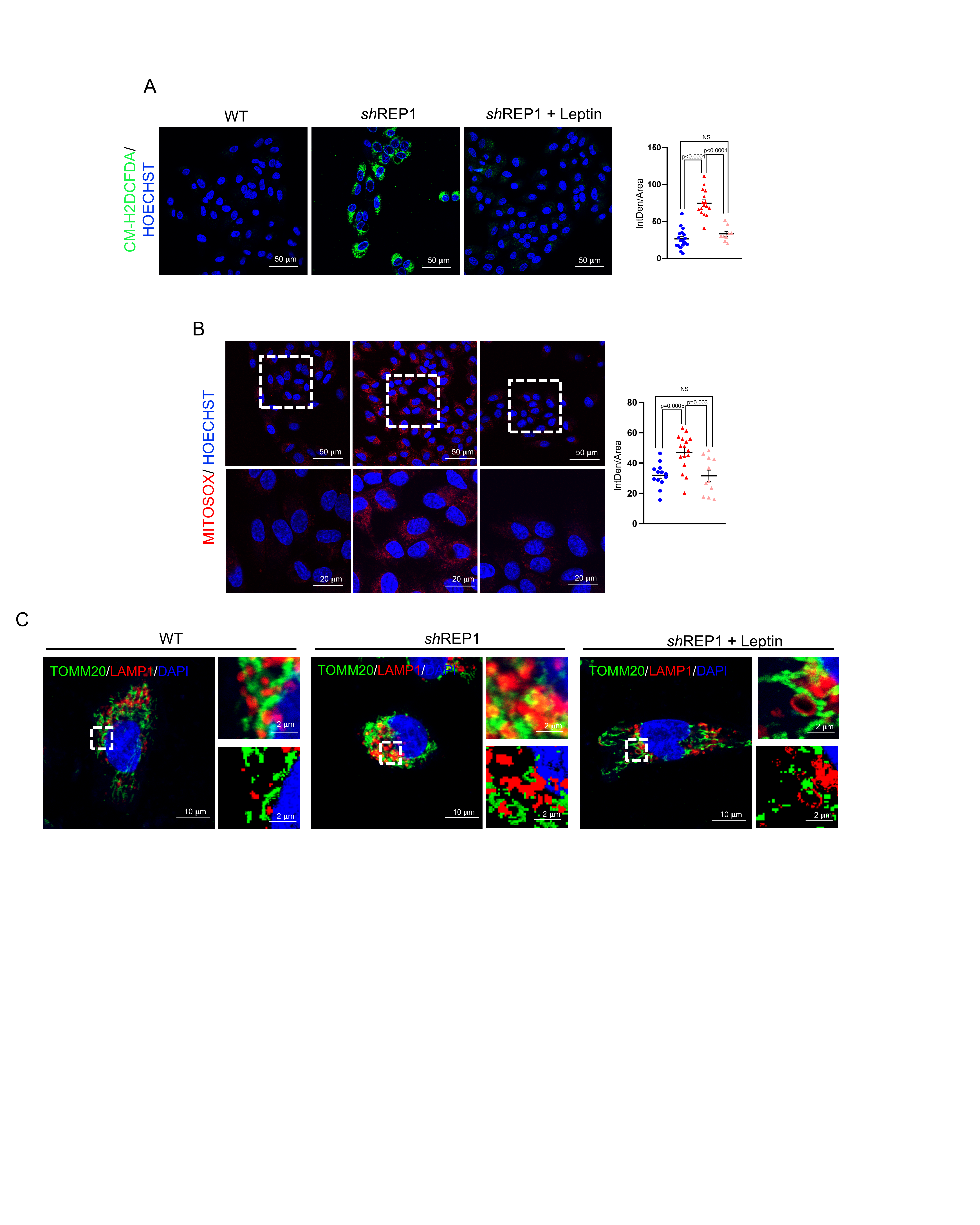

Supplement: Supplementary file 6 — Supplementary Figure 5 [file 41419_2026_8592_MOESM6_ESM.tif]

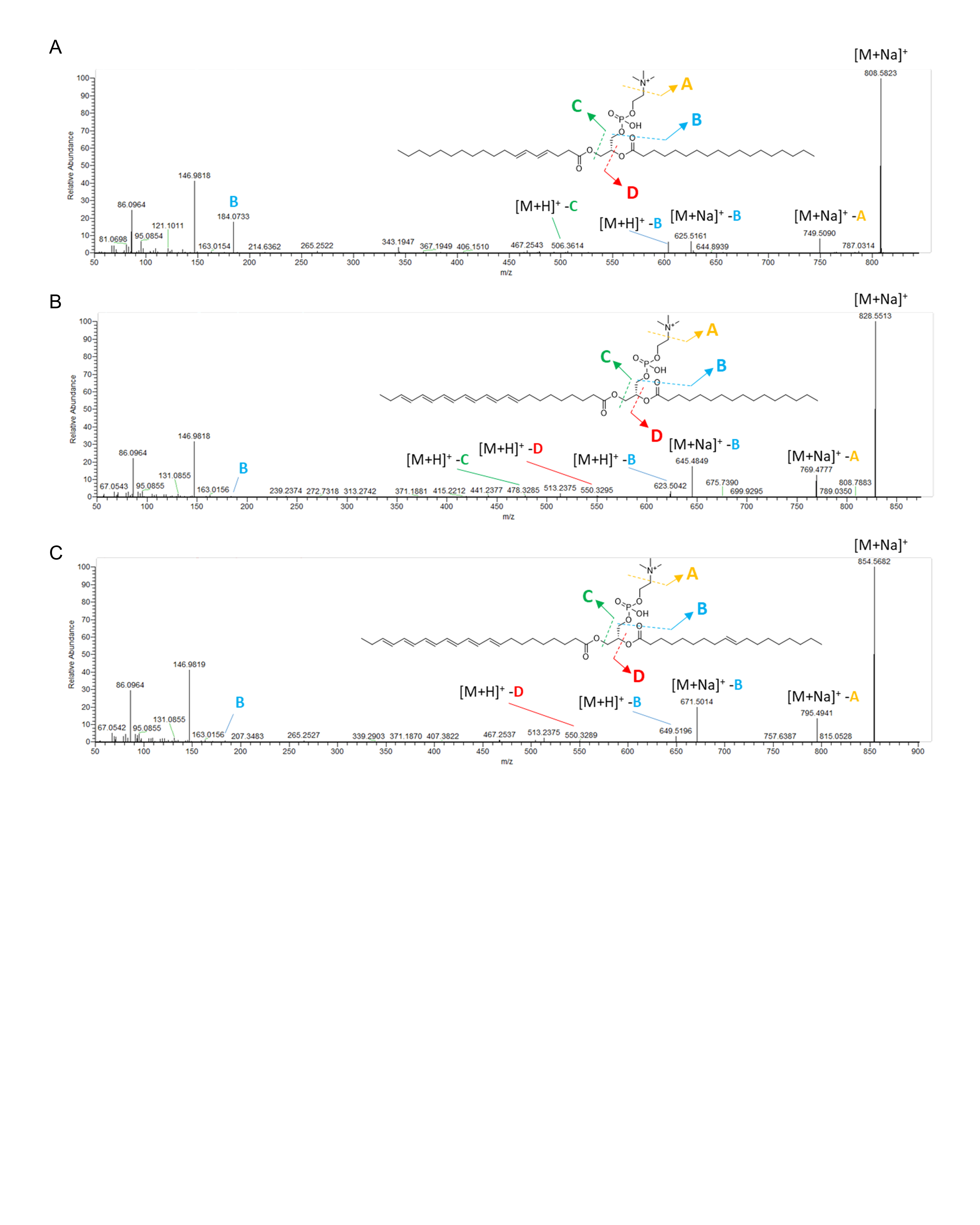

Supplement: Supplementary file 7 — Supplementary Figure 6 [file 41419_2026_8592_MOESM7_ESM.tif]

Figure 1

F

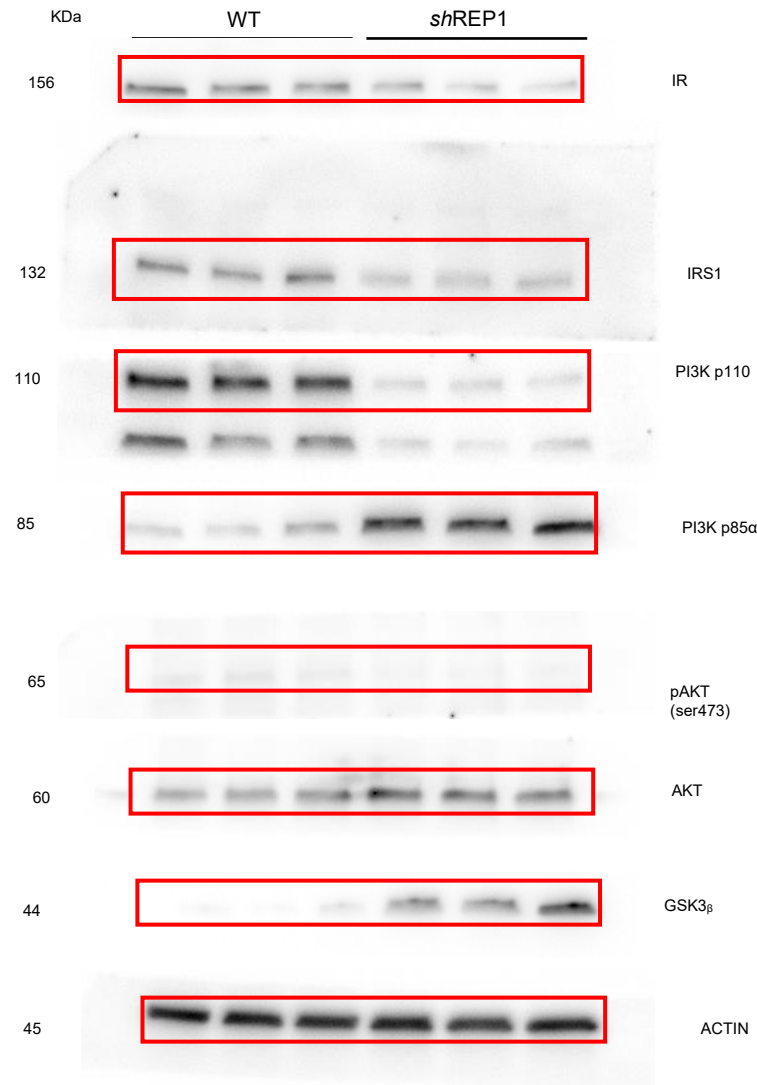

G

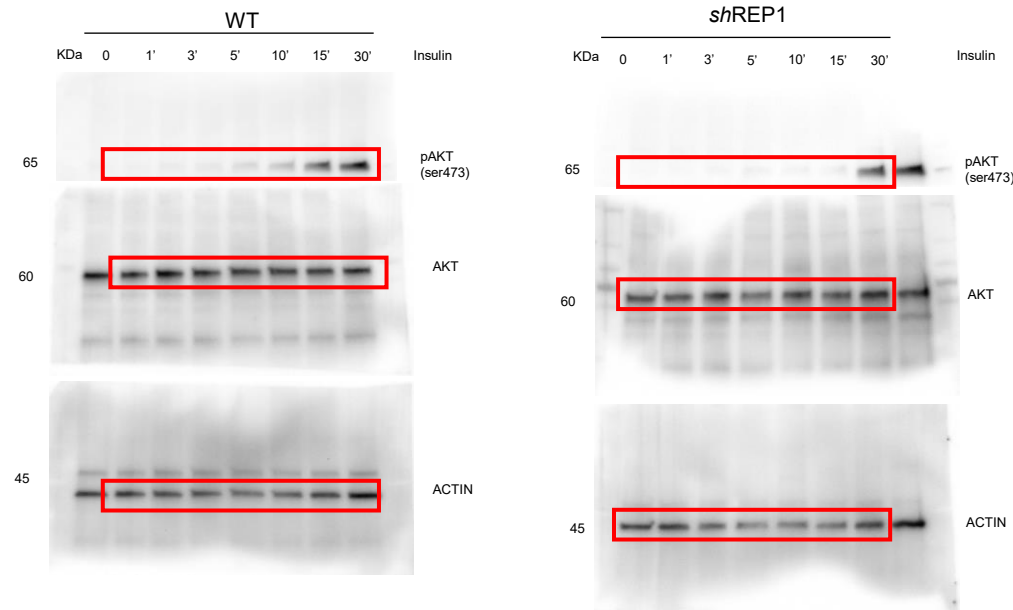

H

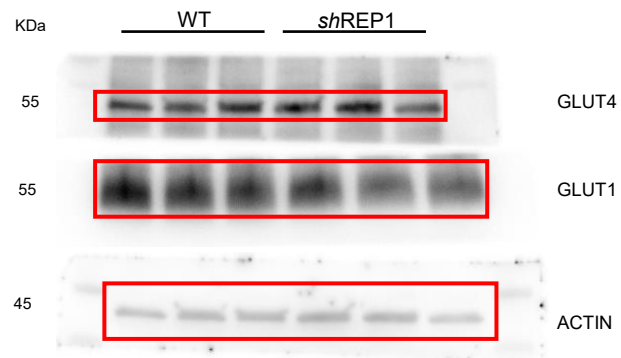

Figure 2 E

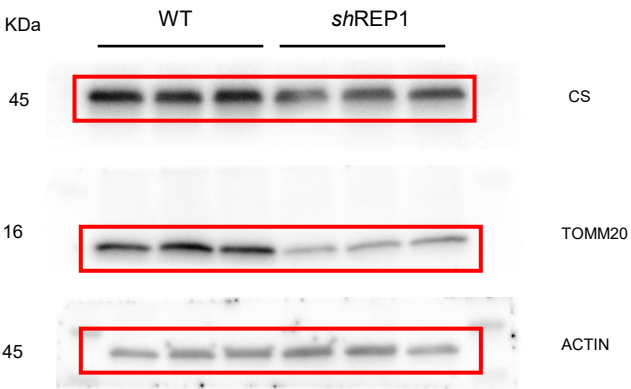

Figure 3 A

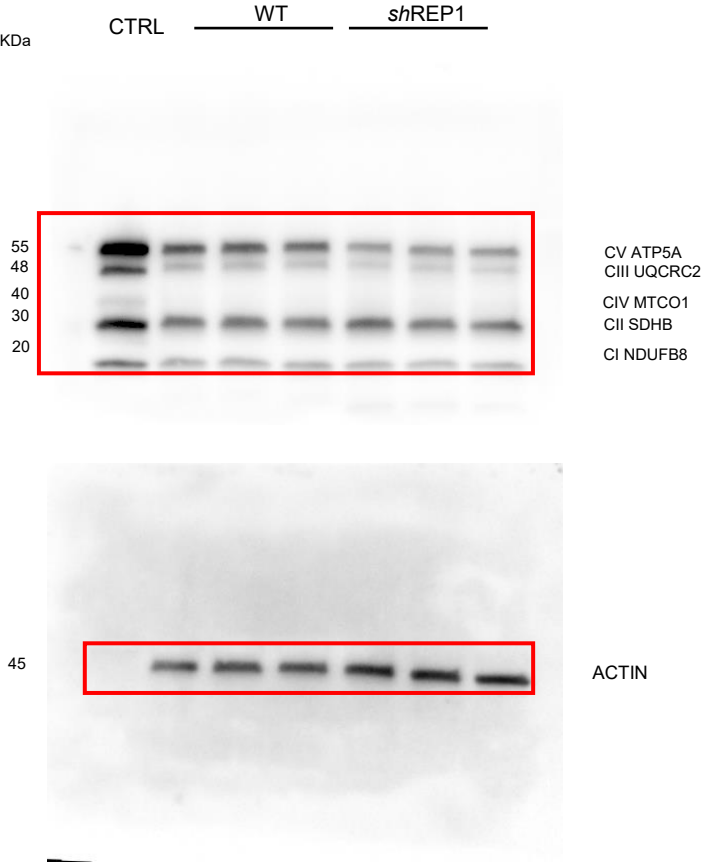

Figure 5 F

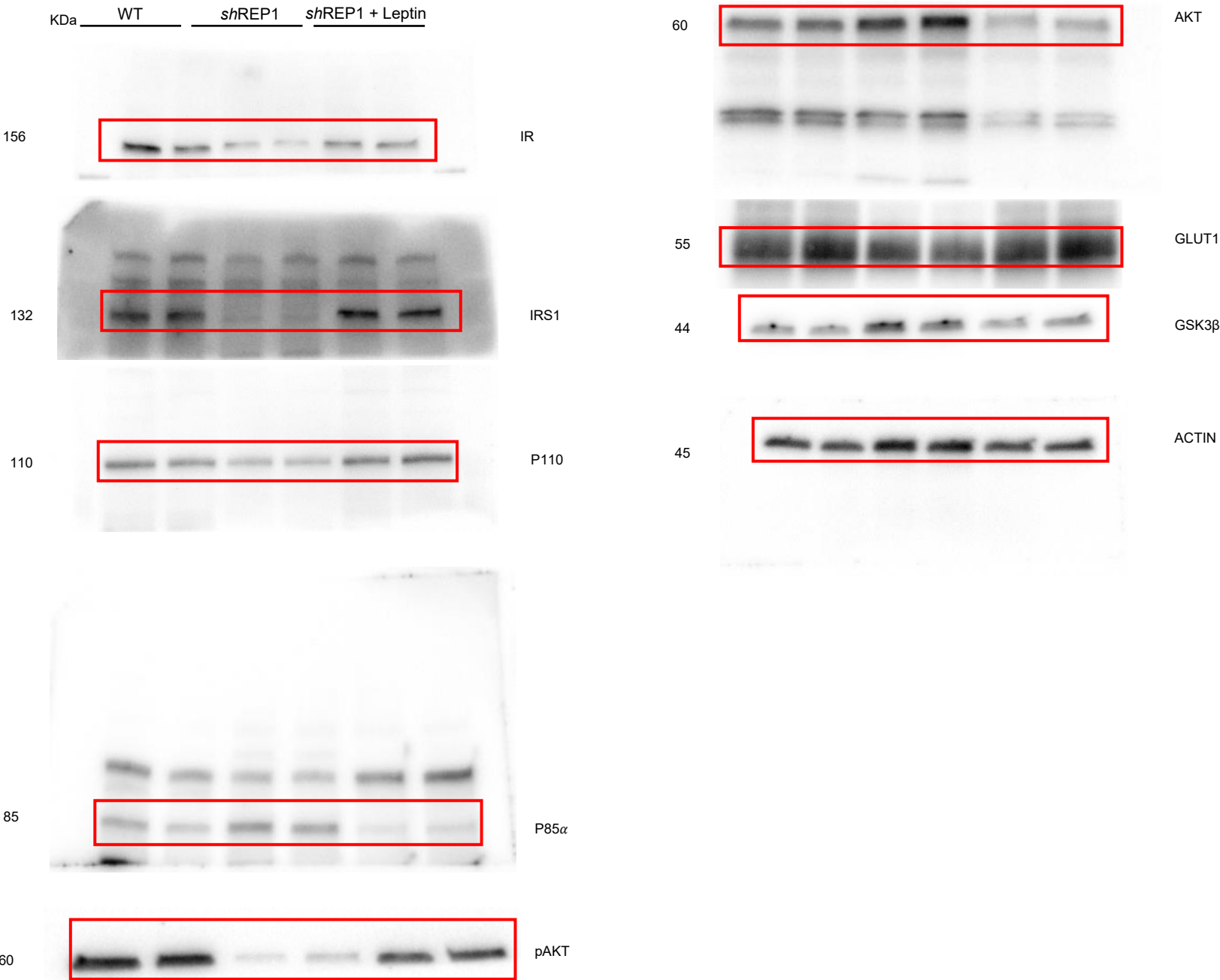

Figure 6

B

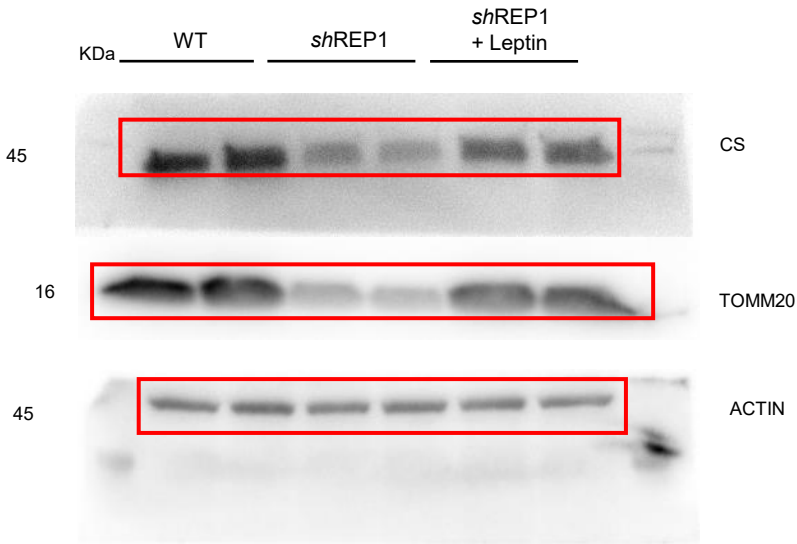

C

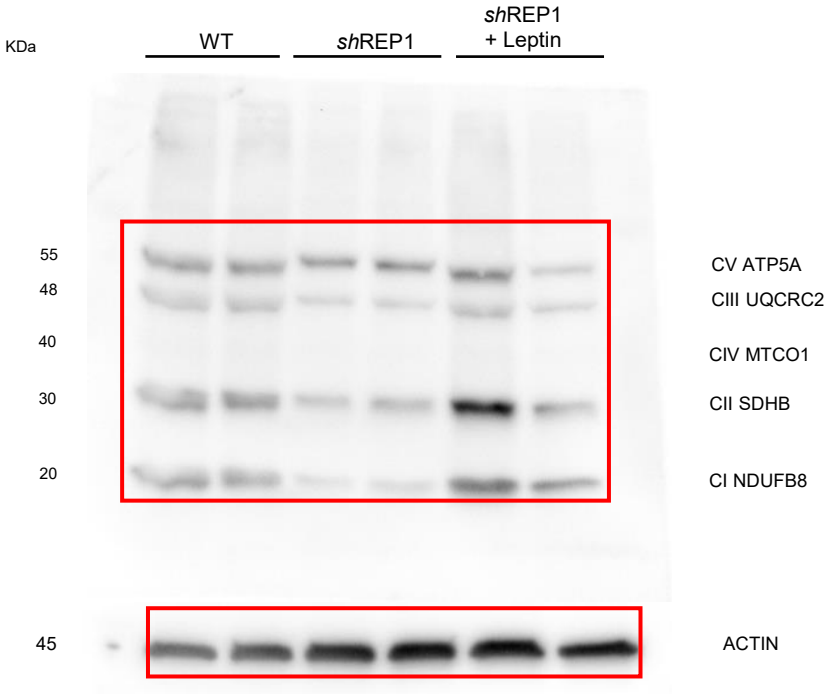

Figure S1

A

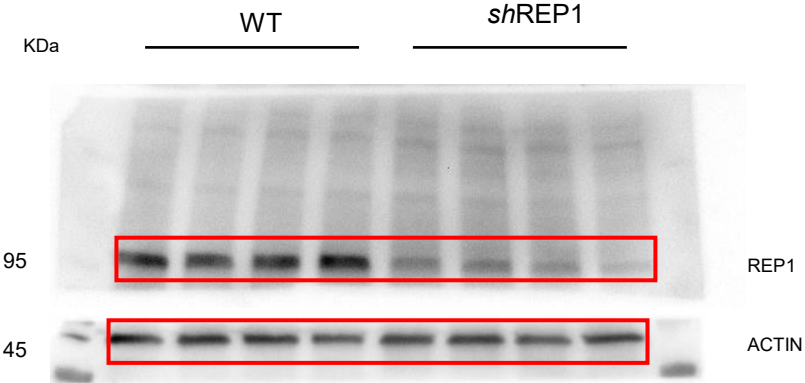

H

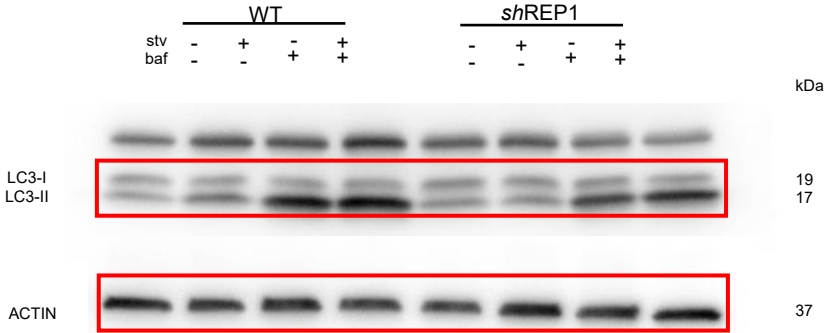

Figure S2

A

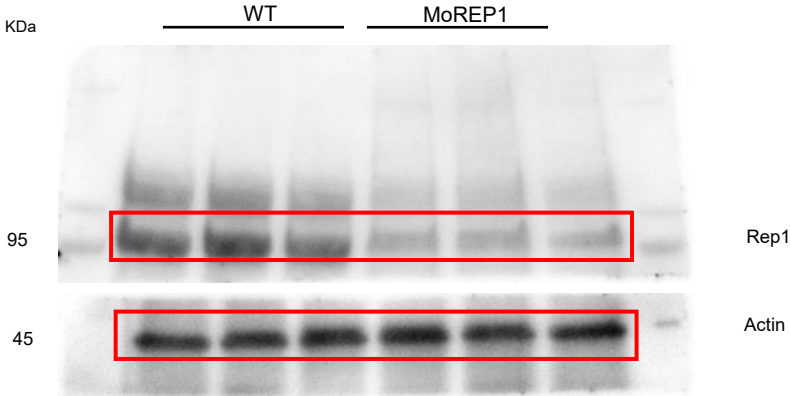

E

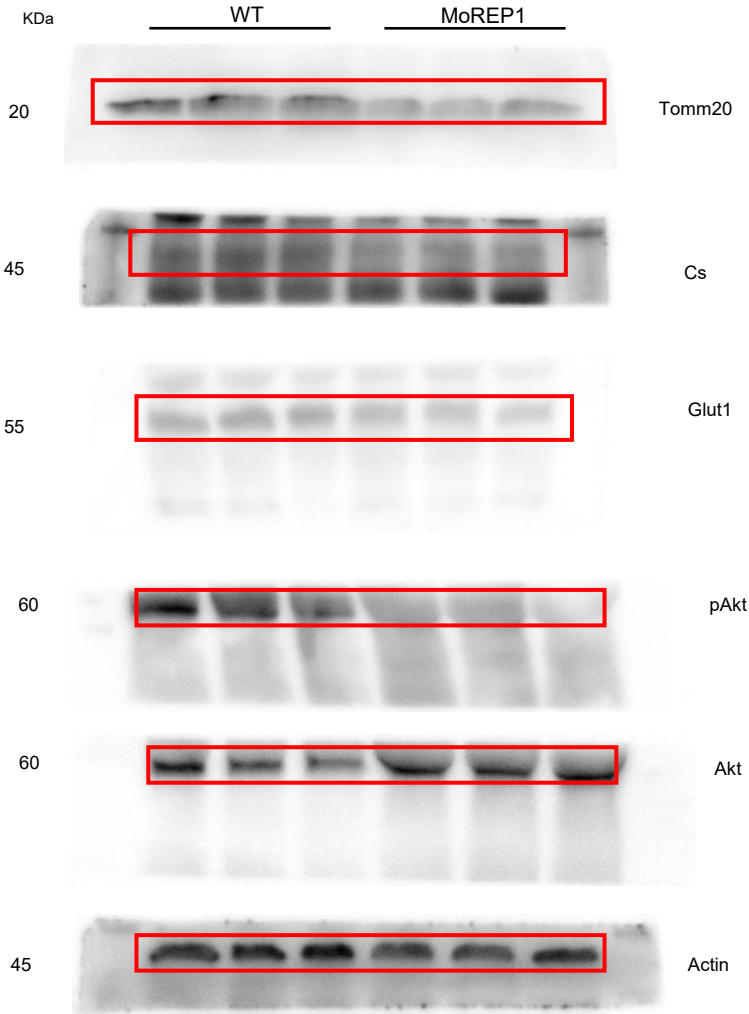

Supplement: Supplementary file 11 — Uncropped WB Data [file 41419_2026_8592_MOESM11_ESM.pdf]
